# Electrophysiology and morphology of human cortical supragranular pyramidal cells in a wide age range

**DOI:** 10.7554/eLife.100390

**Published:** 2025-03-28

**Authors:** Pál Barzó, Ildikó Szöts, Martin Tóth, Éva Adrienn Csajbók, Gábor Molnár, Gábor Tamás

**Affiliations:** 1 Department of Neurosurgery, University of Szeged Szeged Hungary; 2 HUN-REN-SZTE Research Group for Cortical Microcircuits, Department of Physiology, Anatomy and Neuroscience, University of Szeged Szeged Hungary; Johns Hopkins University United States; Brandeis University United States

**Keywords:** cortex, pyramidal cell, ageing, Human

## Abstract

The basic excitatory neurons of the cerebral cortex, the pyramidal cells, are the most important signal integrators for the local circuit. They have quite characteristic morphological and electrophysiological properties that are known to be largely constant with age in the young and adult cortex. However, the brain undergoes several dynamic changes throughout life, such as in the phases of early development and cognitive decline in the aging brain. We set out to search for intrinsic cellular changes in supragranular pyramidal cells across a broad age range: from birth to 85 y of age and we found differences in several biophysical properties between defined age groups. During the first year of life, subthreshold and suprathreshold electrophysiological properties changed in a way that shows that pyramidal cells become less excitable with maturation, but also become temporarily more precise. According to our findings, the morphological features of the three-dimensional reconstructions from different life stages showed consistent morphological properties and systematic dendritic spine analysis of an infantile and an old pyramidal cell showed clear significant differences in the distribution of spine shapes. Overall, the changes that occur during development and aging may have lasting effects on the properties of pyramidal cells in the cerebral cortex. Understanding these changes is important to unravel the complex mechanisms underlying brain development, cognition, and age-related neurodegenerative diseases.

## Introduction

After birth, the brain undergoes developmental changes for a prolonged time that involve a series of complex and accurately orchestrated processes (Rakic, 2009). The production and migration of neurons is largely complete at the beginning of postnatal development, and then the intrauterine developmental processes continue: gray and white matter thickening, myelination, synaptogenesis, pruning, and establishment of the basic anatomical architecture for initial neural pathway function. Subsequently, local connections within cortical circuits are fine-tuned, and increasingly complex, longer-term connections are established between circuits (Stiles and Jernigan, 2010). After that, the changes do not end, but continue throughout human life. They are mostly driven by environmental influences and experiences and lead to changes in metabolic activities (Kuzawa et al., 2014), changes in functional connectivity patterns (Kelly et al., 2009) and, with the maturation of white matter (Beck et al., 2021; Yeatman et al., 2014) changes in the speed of long-distance transmission (van Blooijs et al., 2023). The final phase is aging, where it slowly declines with advancing age, leading to a decline in cognitive signal processing functions and often resulting in neurodegenerative diseases (Peters, 2006). The cortical supragranular glutamatergic cell (or pyramidal cell) provides the excitatory synaptic inputs for local inhibitory circuitry and other pyramidal cells by which they create distinct subnetworks (Yoshimura et al., 2005). The development and formation of dendrites (Petanjek et al., 2008; Koenderink and Uylings, 1995) and synapses (Huttenlocher and Dabholkar, 1997) of pyramidal cells in the human cerebral cortex has been documented to some extent by postmortem studies, besides much less is known about their biophysical maturation and electrical properties in the early stages of development and the subsequent change or maintenance in later ages. Numerous studies demonstrated in non-primate animal models that the electrical characteristics of neurons change prominently in the early postnatal stage (Molnár et al., 2020). Changes in the intrinsic membrane properties (Picken Bahrey and Moody, 2003), the input resistance or the kinetics of the elicited action potentials were reported (Kroon et al., 2019; Elston and Fujita, 2014) in connection with maturation of macaque and rodent pyramidal cells. To date, however, no cross-age studies have been conducted on the electrophysiological parameters of human pyramidal neurons. We have studied in detail the postnatal lifetime profile of the physiological and morphological properties of supragranular (layer 2/3) neurons of human pyramidal cells from neurosurgical resections. To this end, we performed whole-cell patch-clamp recordings and 3D anatomical reconstructions of human cortical pyramidal cells from 109 patients aged 1 m to 85 y for comprehensive data analysis to obtain the morphoelectric lifetime profile of supragranular pyramidal cells.

## Results

### Age-dependent differences in intrinsic subthreshold membrane properties

**Figure 1.**
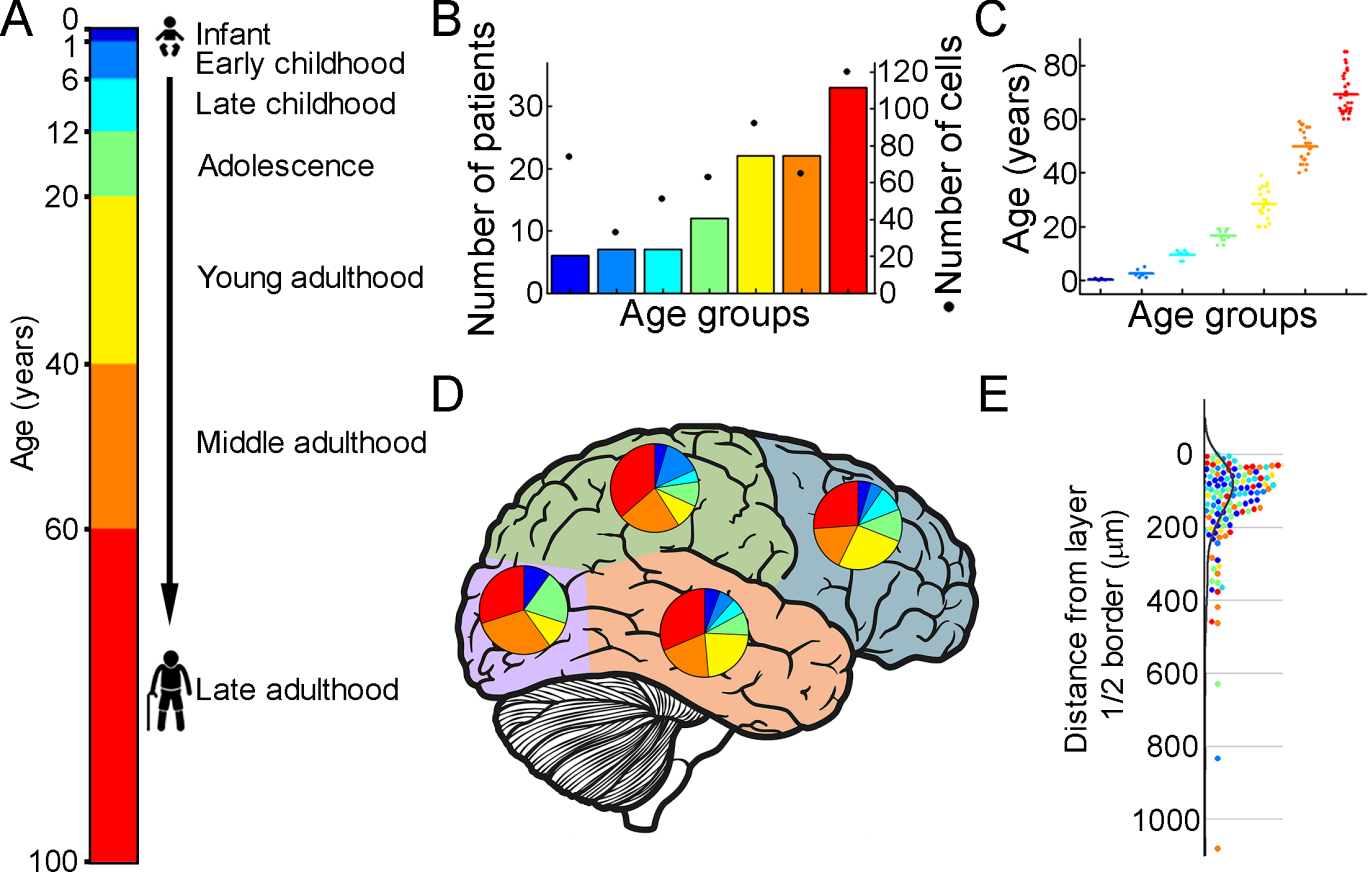
Illustration of the patient data on the samples utilized. (**A**) Illustration of the defined age groups. (**B**) Number of patients involved in age groups, (n=6, 7, 7, 12, 22, 22, 33 from infant to late adulthood, respectively). Dots show the number of human layer 2/3 pyramidal cells in our dataset regarding the defined age groups (n=74, 33, 51, 63, 92, 66, 120 from infant to late adulthood, respectively). (**C**) Distributions of patient ages within age groups. (**D**) Brain model indicates the number of surgically removed tissues from the cortical lobes. Colors indicate age groups. (**E**) The distribution of recovered cell bodies distance from the L1/2 border.

To extract biophysical properties of excitatory cells of human brain specimens we performed whole-cell patch-clamp recordings of pyramidal cells from neurosurgically removed human neocortical tissue sections. The samples were mainly from the frontal and temporal lobes (Figure 1D, Figure 1—figure supplement 1D), mostly from patients with tumors or hydrocephalus (Figure 1—figure supplement 1B, Supplementary file 1). Data were collected from 109 patients aged 1 m to 85 y (Figure 1B and C) from 498 human cortical layer 2/3 (L2/3) pyramidal cells. To confirm that the studied pyramidal cells originate from the L2/3, we measured the distance between the cell body and the L1 border (Berg et al., 2021). 36% of the cells recovered their soma, with a distance of 129.69±130.77 μm from the L1 border (Figure 1E). The data set was divided into seven age groups (Kang et al., 2011; Bethlehem et al., 2022): infant:<1 y, early childhood: 1–6 y, late childhood: 7–12 y, adolescence: 13–19 y, young adulthood: 20–39 y, middle adulthood: 40–59 y, late adulthood: ≥60 y (Figure 1A).

**Figure 1—figure supplement 1.**
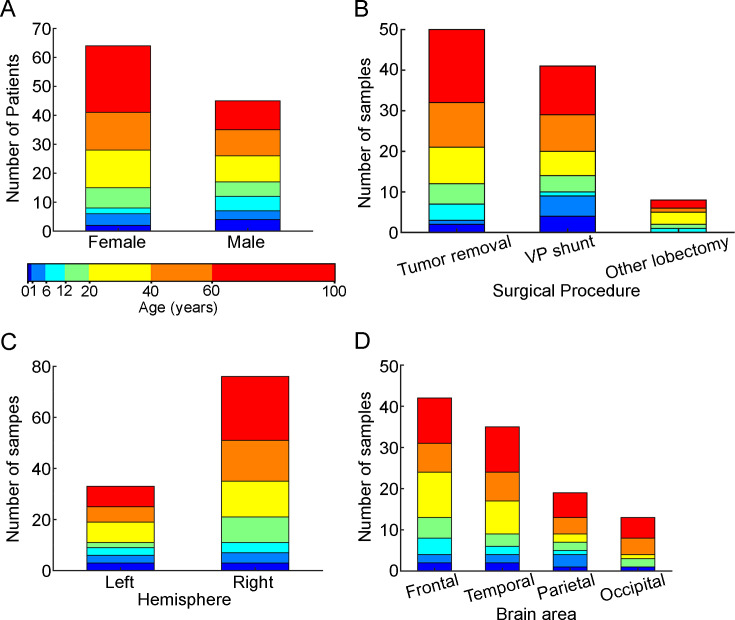
Patient metadata. (**A**) Distribution of patients by gender (female: infant n=2, early childhood n=4, late childhood n=2, adolescence n=7, young adulthood n=13, middle adulthood n=13, late adulthood n=23, male: infant n=4, early childhood n=3, late childhood n=5, adolescence n=5, young adulthood n=9, middle adulthood n=9, late adulthood n=10), (**B**) surgical procedure (tumor removal: infant n=2, early childhood n=2, late childhood n=5, adolescence n=7, young adulthood n=13, middle adulthood n=12, late adulthood n=19, VP (ventriculoperitoneal) shunt: infant n=4, early childhood n=5, late childhood n=1, adolescence n=4, young adulthood n=6, middle adulthood n=9, late adulthood n=12, other: infant n=0, early childhood n=0, late childhood n=1, adolescence n=1, young adulthood n=3, middle adulthood n=1, late adulthood n=2), (**C**) hemisphere (left: infant n=3, early childhood n=3, late childhood n=3, adolescence n=2, young adulthood n=8, middle adulthood n=6, late adulthood n=8, right: infant n=3, early childhood n=4, late childhood n=4, adolescence n=10, young adulthood n=14, middle adulthood n=16, late adulthood n=25) and (**D**) brain area (frontal: infant n=2, early childhood n=2, late childhood n=4, adolescence n=5, young adulthood n=11, middle adulthood n=7, late adulthood n=11, temporal: infant n=2, early childhood n=2, late childhood n=2, adolescence n=3, young adulthood n=8, middle adulthood n=7, late adulthood n=11, parietal: infant n=1, early childhood n=3, late childhood n=1, adolescence n=2, young adulthood n=2, middle adulthood n=4, late adulthood n=6, occipital: infant n=1, early childhood n=0, late childhood n=0, adolescence n=2, young adulthood n=1, middle adulthood n=4, late adulthood n=5). Stacked columns are colored regarding the age groups shown on the colorbar.

**Figure 2.**
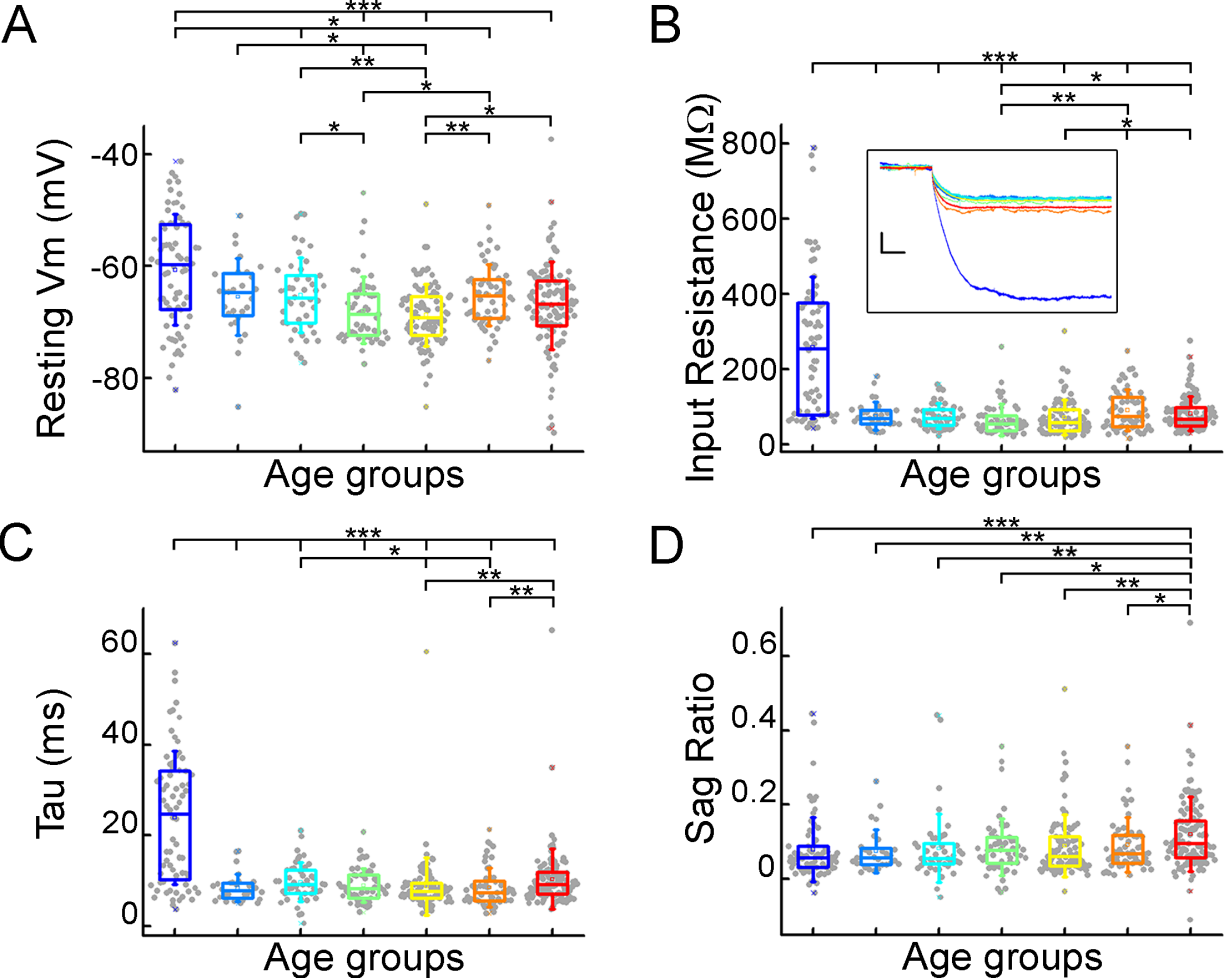
Subthreshold membrane properties vary across life stage. (**A–D**) Boxplots show resting membrane potential (**A**), input resistance (**B**), tau (**C**), and sag ratio (**D**) distributions in various age groups. Inset shows representative voltage traces from each group. Scale bar: 5 mV; 20 ms. Asterisks indicate significance (Kruskal–Wallis test with post-hoc Dunn test, *p<0.05, **p<0.01, ***p<0.001).

We evaluated the voltage deflections induced by negative and positive current injections and extracted subthreshold membrane features such as resting membrane potential, input resistance, time constant (tau), and sag ratio in 457 cortical pyramidal cells from 99 patients. We found that the subthreshold features from samples of infant significantly different from those from other age groups (Figure 2A, B, C and D; resting membrane potential: p=3.53 × 10^–8^, input resistance: p=1.29 × 10^–16^, tau: p=1.31 × 10^–15^, sag ratio: p=5.2 × 10^–4^, Kruskal-Wallis test) (Figure 2—figure supplement 1). The resting membrane potential was significantly more positive in the first year of life than in the other age groups. Before adulthood, a slight decrease was observed in the resting membrane potential across the groups (Table 1; Figure 2A). Input resistance, tau and sag ratio were measured on voltage deflections elicited by injecting negative (–100 pA) current steps into the cells. A significant decrease in input resistance (Table 1) was observed with the largest reduction after the first year of age (p=1.88 × 10^–6^, Kruskal–Wallis test with post-hoc Dunn test) (Figure 2B). The membrane time constant also decreased significantly in the older groups compared to the infant group, after infancy we found more conserved mean values of membrane time constant into older age (Table 1; Figure 2C). The ratio of the maximal deflection and the steady-state membrane potential during a negative current step (sag ratio) is significantly higher in late adulthood than in the early stages of life (Table 1; Figure 2D). Note that the high variance of the infant group data (e.g. resting membrane potential, input resistance, tau) are due to the dynamic change over the 0–1 y period (Figure 2—figure supplement 2).

**Figure 2—figure supplement 1.**
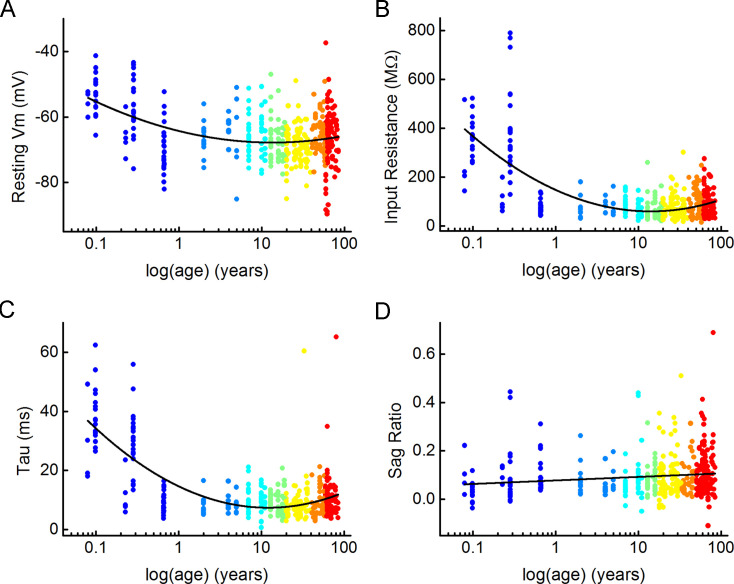
Distribution of subthreshold electrophysiological features with age. (**A–D**) Scatter plots showing the passive electrophysiological characteristics: resting membrane potential (**A**), input resistance (**B**), tau (**C**), and sag ratio (**D**) throughout the lifespan. Dots are colored according to the age groups, age is represented in years on a logarithmic scale.

**Figure 2—figure supplement 2.**
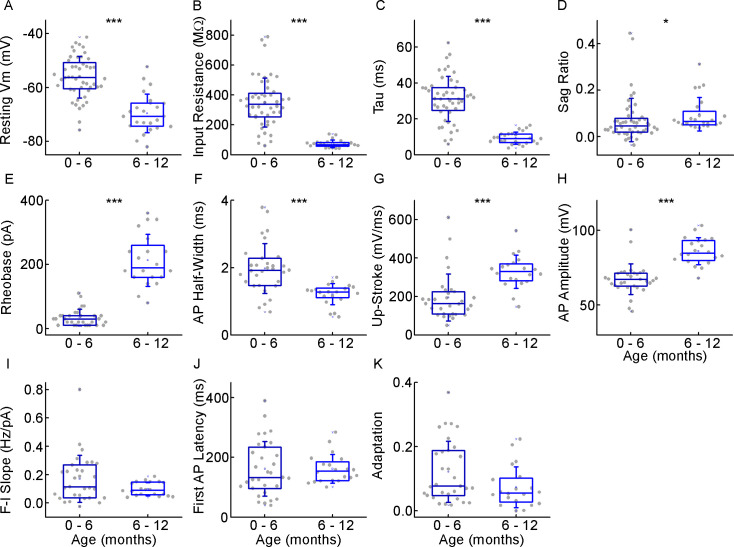
Electrophysiological differences during the first year of life. (**A–D**) Boxplots showing differences in passive properties, resting membrane potential (**A**), input resistance (**B**), tau (**C**), and sag ratio (**D**) within the infant age group (*p<0.05, **p<0.01, ***p<0.001, two-sample t-test (**A, B, C**) or Mann-Whitney test (**D**)). (**E–H**) Differences in the action potential kinetics, rheobase (**E**), action potential (AP) half-width (**F**), up-stroke (**G**), and AP amplitude (**H**) between the cells from the first and the second half of the first year of life. Asterisks indicate significance (*p<0.05, **p<0.01, ***p<0.001, Mann-Whitney test). (**I–K**) Deviations in AP firing pattern parameters, F-I slope (**H**), first AP latency (**I**), and adaptation (**J**) during infancy.

**Table 1.** Subthreshold membrane properties.

Subthreshold properties	InfantN=72	Early childhoodN=28	Late childhoodN=45	AdolescenceN=54	Young adulthoodN=89	Middle adulthoodN=56	LateAdulthoodN=113
	Mean ± SD	Mean ± SD	Mean ± SD	Mean ± SD	Mean ± SD	Mean ± SD	Mean ± SD
**Resting Vm (mV**)	–60.64±9.86	–65.44±6.82	–65.17±6.68	–67.86±5.94	–68.69±5.54	–65.14±5.5	–67.05±7.86
**Input resistance (MΩ**)	257.25±188.06	75.27±37.5	74.61±34.29	64.79±41.82	70.45±46.9	90.14±54.37	81.14±46.36
**Tau (ms**)	23.88±14.7	8.49±3.08	9.73±4.24	8.99±3.71	8.76±6.33	8.53±4.24	10.39±6.63
**Sag ratio**	0.079±0.87	0.075±0.058	0.082±0.092	0.086±0.076	0.09±0.084	0.092±0.073	0.12±0.1

### Suprathreshold properties across age-groups

**Figure 3.**
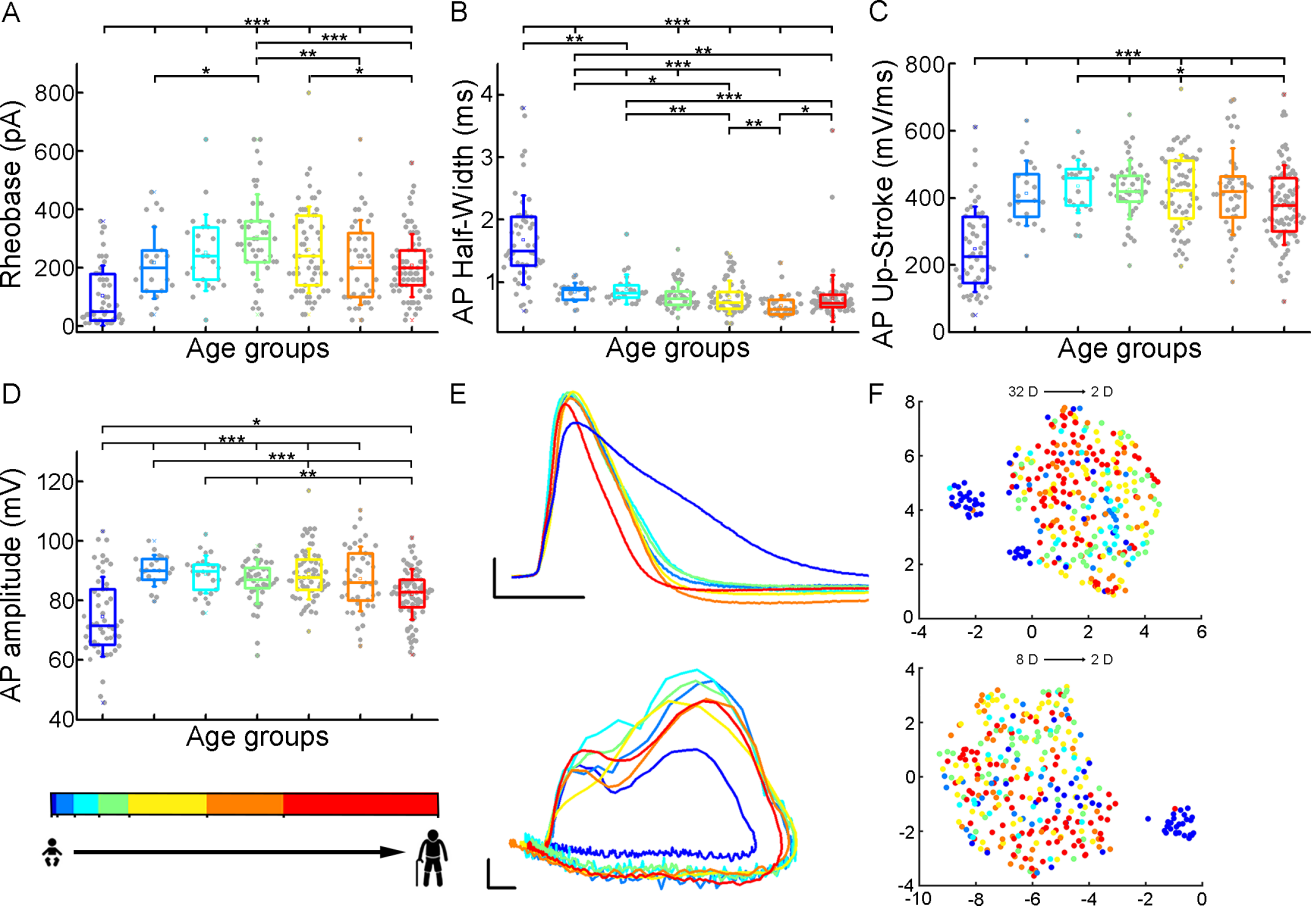
Age-related differences in the action potential kinetics. (**A–D**) Boxplots show differences in rheobase (**A**), action potential half-width (**B**), action potential up-stroke (**C**), and action potential amplitude (**D**) between the age groups. Asterisks indicate statistical significance (*p<0.05, **p<0.01, ***p<0.001). (**E**) Representative action potentials aligned to threshold potential onset (scale: x-axis: 1 ms, y-axis: 20 mV) (top) and phase plots of the representative action potentials (APs) (scale: x-axis: 10 mV, y-axis: 100 mV/ms) (bottom). (**F**) Uniform Manifold Approximation and Projection (UMAP) of 32 (Table 4) (top) and eight selected electrophysiological properties (resting Vm, input resistance, tau, sag ratio, rheobase, AP half-width, AP up-stroke, and AP amplitude) (bottom) with data points for 331 cortical L2/3 pyramidal cells, colored with the corresponding age groups.

To initiate action potentials we injected positive current steps increased by 20 pA into the cells and recorded various types of input-output transformations. We extracted 25 features from different action potentials (AP) and firing patterns and assessed active membrane properties from recordings filtered for appropriate electrophysiological quality (see Methods). We found that the infant group differs most from the other age groups in several of the suprathreshold properties (Figure 3), but other trends are also apparent (rheobase current: p=8.71 × 10^–12^, AP half-width: p=9.57 × 10^–25^, AP up-stroke velocity: p=1.63 × 10^–12^, AP amplitude: p=2.24 × 10^–11^, Kruskal-Wallis test) (Figure 3—figure supplement 1). For example, the average rheobase current, the minimum current that can trigger an action potential, is significantly lower in the early ages of life than in data collected from adolescence stage (Table 2) (infant vs. adolescence p=4.15 × 10^–13^, Kruskal–Wallis test with post-hoc Dunn test). Further on the age scale, we found that rheobase current was increased with age reaching a maximum value at the adolescent age and then declining to a significantly lower level (adolescent vs. late adulthood, p=5.23 × 10^–4^, Kruskal–Wallis test with post-hoc Dunn test, Figure 3A). The AP half-width averages among a declining trend through the groups of age (Table 2) varied considerably across age groups forming significant differences between childhood and adulthood ages (Figure 3B). Action potential up-stroke velocities were significantly slower in the infant APs than in all the other ages (Table 2; Figure 3C). The amplitude of the elicited APs also showed age-dependent differences between age groups (Table 2); in the first year of life, the amplitude of APs were significantly lower than in other age groups. In adulthood, a significant decrease with age was observed (Figure 3D). Electrophysiological differences markedly separated the infant group from the older age groups shown on UMAP projection (Figure 3F, Figure 3—figure supplement 2).

**Figure 3—figure supplement 1.**
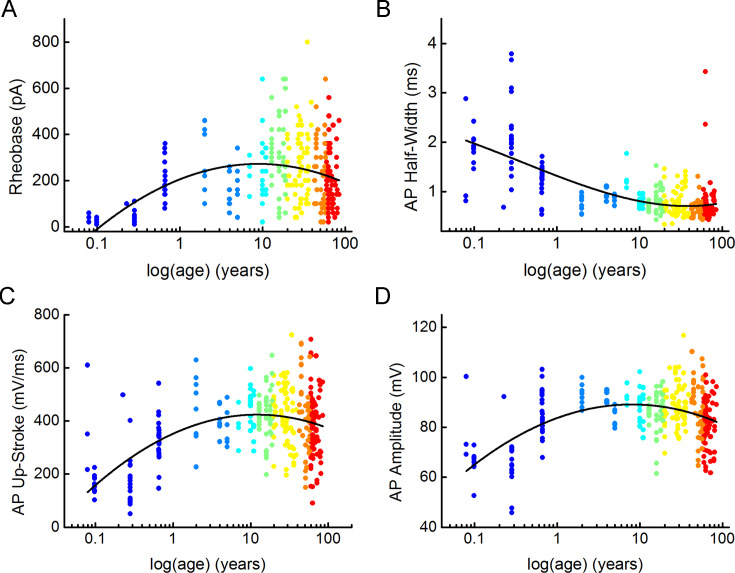
Distribution of suprathreshold electrophysiological features with age. (**A–D**) Diagrams show changes through age in rheobase (**A**), action potential half-width (**B**), action potential up-stroke (**C**), and action potential amplitude (**D**). Dots are colored according to the age groups, age is represented in years on a logarithmic scale.

**Figure 3—figure supplement 2.**
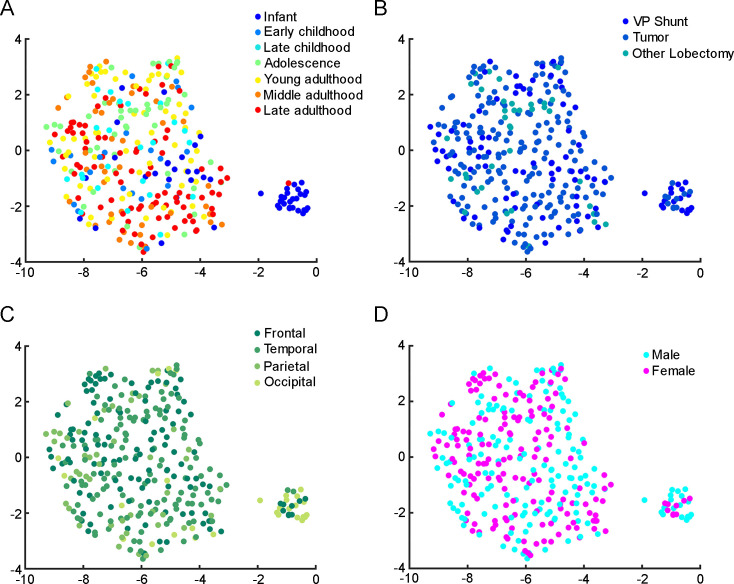
Relationship between patient metadata and electrophysiology. Uniform Manifold Approximation and Projection (UMAP) of eight electrophysiological properties (resting Vm, input resistance, tau, sag ratio, rheobase, AP half-width, AP up-stroke, and AP amplitude) with data points for 331 cortical L2/3 pyramidal cells, colored with the corresponding age groups (**A**), surgical procedures (**B**), brain area (**C**), and gender (**D**).

**Table 2.** Action potential and firing pattern parameters.

Suprathreshold properties	InfantN=51	Early childhoodN=21	Late childhoodN=25	AdolescenceN=45	Young adulthoodN=63	Middle adulthoodN=43	LateAdulthoodN=83
	Mean ± SD	Mean ± SD	Mean ± SD	Mean ± SD	Mean ± SD	Mean ± SD	Mean ± SD
**Rheobase (pA**)	104.51±103.18	218.1±123.27	252.8±131.17	306.22±147.21	262.9±148.06	219.07±145.52	207.71±107.83
**AP half-width (ms**)	1.68±0.71	0.84±0.15	0.88±0.24	0.78±0.22	0.76±0.27	0.62±0.17	0.74±0.38
**AP up-stroke (mV/ms**)	247.43±127.27	413.68±97.11	434.75±78.32	424.69±87.58	418.49±110.33	419.15±129.29	379.22±118.45
**AP amplitude (mV**)	74.62±13.4	90±5.23	88.82±6.35	86.31±7.36	88.81±8.52	87.28±10.77	82.02±8.49
**F-I slope (Hz/pA**)	0.142±0.137	0.152±0.066	0.144±0.071	0.139±0.141	0.127±0.08	0.176±0.113	0.165±0.117
**First AP latency (ms**)	161.26±76.81	121.36±76.88	115.29±81.27	136.69±109.78	132.42±84.68	140.61±143.43	106.39±52.31
**Adaptation**	0.103±0.087	0.061±0.062	0.068±0.06	0.105±0.1	0.119±0.118	0.114±0.153	0.124±0.1

**Figure 4.**
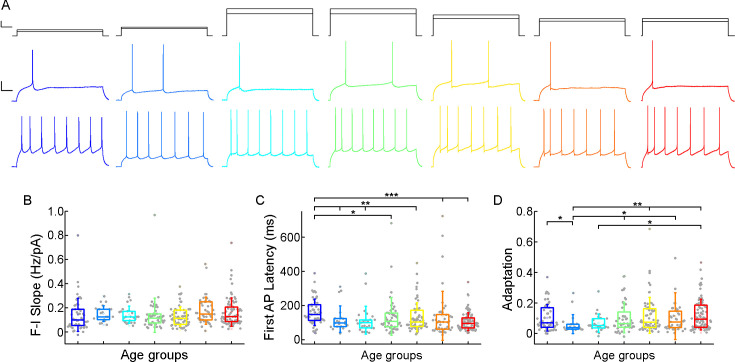
Age-dependency of the action potential (AP) firing pattern parameters. (**A**) Representative membrane potential responses to an 800 ms long rheobase (middle) (left to right: infant, early childhood, late childhood, adolescence, young adulthood, middle adulthood, late adulthood), and increased current steps (bottom) colored respectively to the age groups. Scale bar top: 1 ms, 100 pA, bottom: 1 ms, 20 mV. (**B–D**) Boxplots show changes across the age groups in f-I slope (**B**), first AP latency (**C**), and adaptation of APs (**D**). Asterisks indicate statistical significance (*p<0.05, **p<0.01, ***<0.001).

Next, we investigated how somatic current inputs are transformed to action potential output by evaluating the firing patterns of cells evoked by injecting prolonged positive current steps (Figure 4A; Figure 4—figure supplement 1). The neurons (n=331) were regular spiking cells with moderate adaptation. The slope of the firing frequency versus the current curve (f-I slope) showed no significant difference across the age groups (p=0.055, Kruskal-Wallis test) (Table 2; Figure 4B). Age-related difference was observed in the latency of the first AP during rheobase current injection (first AP latency), in the first year of life the latency of the first spike is significantly higher than all the other groups of age (p=7.67 × 10^–4^, Kruskal-Wallis test), which is the most prominent with the oldest age group (p=8.41 × 10^–6^, Kruskal–Wallis test with post-hoc Dunn test) (Table 2; Figure 4C). The adaptation of the AP frequency response to the same current injection stimulus also showed differences (p=0.032, Kruskal-Wallis test) between the younger and the groups older than 13 y patients, we found the lowest adaptation values in early childhood (Table 2; Figure 4D).

**Figure 4—figure supplement 1.**
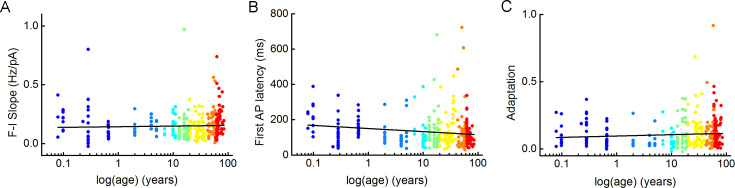
Distribution of firing pattern characteristics with age. (**A–C**) Plots show F-I slope (**A**), first action potential (AP) latency (**B**), and adaptation of APs (**C**) depending on age. Dots are colored according to the age groups, age is represented in years on a logarithmic scale.

**Figure 4—figure supplement 2.**
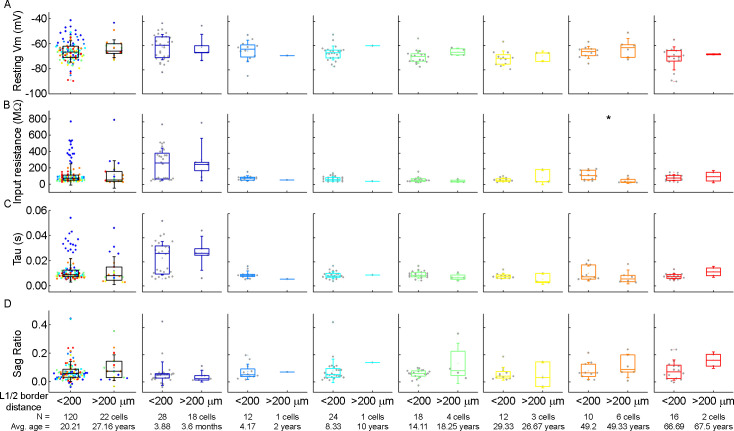
Comparison of the subthreshold properties of the cells as a function of their distance from the layer border. (**A–D**) Boxplots show resting membrane potential (**A**), input resistance (**B**), tau (**C**), and sag ratio (**D**) of the pyramidal cells whose soma is located at a distance greater than and less than 200 μm from the L1 border. From left to right: data from all age groups, infant, early childhood, late childhood, adolescence, young adulthood, middle adulthood, and late adulthood groups. Asterisks indicate significance (*p<0.05, Mann-Whitney test).

**Figure 4—figure supplement 3.**
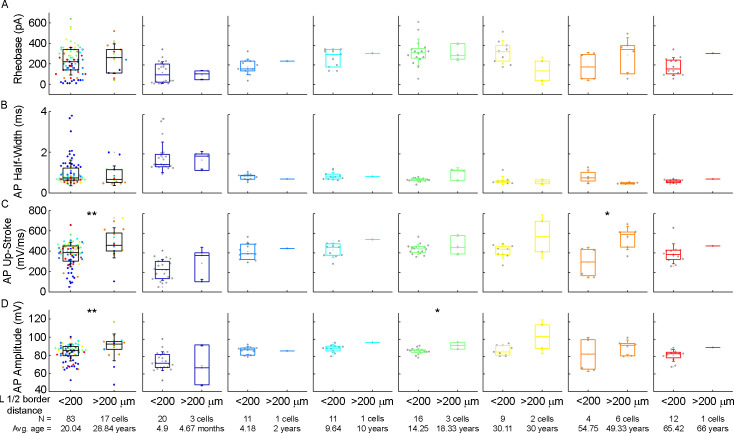
Comparison of the action potential properties of the cells as a function of their distance from the layer border. (**A–C**) Boxplots show rheobase (**A**), action potential half-width (**B**), action potential upstroke (**C**), and action potential amplitude (**D**) of cells whose soma is located at a distance greater than and less than 200 μm from the L1 border. From left to right: data from all age groups, infant, early childhood, late childhood, adolescence, young adulthood, middle adulthood, and late adulthood. Asterisks indicate significance (*p<0.05, **p<0.01, Mann-Whitney test).

**Figure 4—figure supplement 4.**
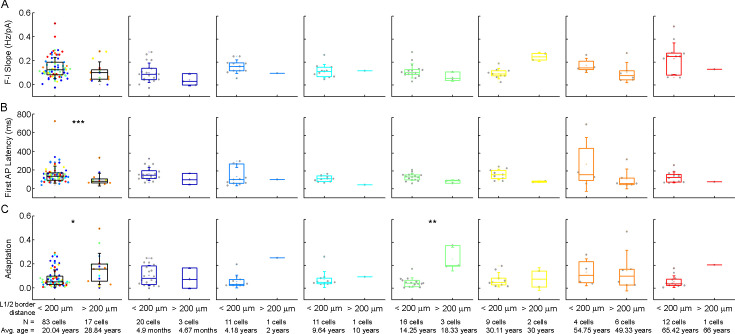
Comparison of the firing pattern characteristics of the cells as a function of their distance from the layer border. (**A–C**) Boxplots show: F-I slope (**A**), latency of the first action potential (AP) (**B**), and adaptation of APs (**C**) of cells whose soma is located at a distance greater than and less than 200 μm from the L1 border. From left to right: data from all ages, infancy, early childhood, late childhood, adolescence, young adulthood, middle adulthood, and late adulthood. Asterisks indicate significance (*p<0.05, **p<0.01, Mann-Whitney test).

Previous research has shown that the biophysical properties of the human pyramidal cells show depth-related correlations throughout the L2/3 (Berg et al., 2021; Kalmbach et al., 2018; Moradi Chameh et al., 2021). Although there are some deeper cells in our dataset, the majority comes from the upper region of the L2/3. We compared the electrophysiological characteristics according to their depth from the border of L1 and L2 to exclude the possibility that the biophysical differences we found were a result of depth dependence. We did not find any overall differences related to distance of the soma from the L1 border within the age groups with a few exceptions. For example, the values of input resistance (p=0.02, Mann-Whitney test) and AP up-stroke velocity (p=0.04, Mann-Whitney test) differ significantly in the middle adulthood group. We found a significant difference in AP amplitude (p=0.02, Mann-Whitney test) and adaptation (p=0.009, Mann-Whitney test) in the adolescence age group (Figure 4—figure supplement 2, Figure 4—figure supplement 3 and Figure 4—figure supplement 4).

### Morphological features of layer 2/3 pyramidal cells in different stages of life

**Figure 5.**
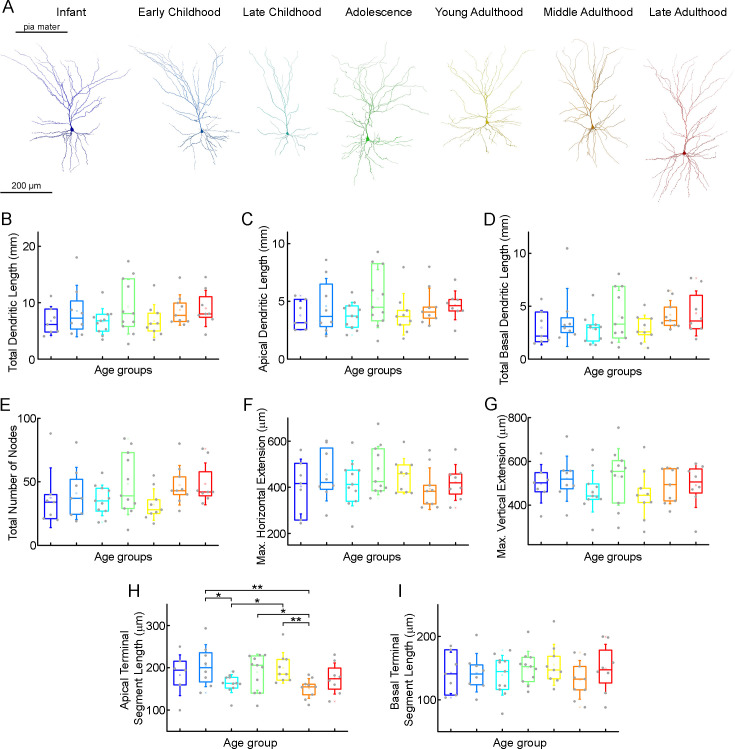
Morphological features of layer 2/3 pyramidal cells in different stages of life. (**A**) Representative reconstructions of L2/3 pyramidal cells (from left to right) from infant (n=7), early childhood (n=8), late childhood (n=11), adolescence (n=11), young adulthood (n=9), middle adulthood (n=9), and late adulthood (n=8) patients. (**B–I**) Boxplots show summarized data from all the reconstructed cells (Figure 4—figure supplement 2) of total dendritic length (**B**), apical dendritic length (**C**), total basal dendritic length (**D**), the total number of nodes on the apical and basal dendrites (**E**), the maximal horizontal (**F**), and the maximal vertical (**G**) extension of dendrites, the average length of the apical (**H**), and basal (**I**) terminal dendritic segments. Asterisks indicate statistical significance (*p<0.05, **p<0.01, Kruskal-Wallis test with post-hoc Dunn test).

To investigate possible morphological differences between the age groups, we filled the pyramidal cells with biocytin during recordings. Only neurons with no signs of deterioration and with complete apical dendrites and no signs of truncated dendritic branches or tufts were considered for morphological analysis. 63 pyramidal cells (Figure 5—figure supplement 1) were reconstructed in 3D at ages 0–73 y (infant n=7, early childhood n=8, late childhood n=11, adolescence n=11, young adulthood n=9, middle adulthood n=9, late adulthood n=8) (Figure 5—figure supplements 2 and 3). Figure 5A shows examples of the reconstructed pyramidal cells. We did not detect significant change in total dendritic length (p=0.37, Kruskal-Wallis test) (Table 3; Figure 5B), apical dendritic length (p=0.6, Kruskal-Wallis test) (Table 3; Figure 5C), or basal dendritic length (p=0.28, Kruskal-Wallis test) (Table 3; Figure 5D) at different ages. To investigate dendritic complexity we measured the total number of dendritic branching and found no significant developmental changes (p=0.18, Kruskal-Wallis test) (Table 3; Figure 5E). We also did not observe significant differences in the size of dendritic branching when we measured the maximum horizontal (p=0.64, Kruskal-Wallis test) (Table 3; Figure 5F) and vertical (p=0.51, Kruskal-Wallis test) (Table 3; Figure 5G) extent of the reconstructed cells. We found significant differences in the average length of the cut terminal segments of apical (p=0.033, Kruskal-Wallis test) (Table 3; Figure 5H) but not of the basal dendrites (p=0.85, Kruskal-Wallis test) (Table 3; Figure 5I) of the cells across the age groups .

**Table 3.** Morphological characteristics across the age groups.

Morphological properties	InfantN=7	Early childhoodN=8	Late childhoodN=11	AdolescenceN=11	Young adulthoodN=9	Middle adulthoodN=9	Late adulthoodN=8
	Mean ± SD	Mean ± SD	Mean ± SD	Mean ± SD	Mean ± SD	Mean ± SD	Mean ± SD
**Total dendritic length (mm**)	6.74±2.56	8.5±4.56	6.62±2.29	9.27±4.82	6.76±2.9	8.7±2.7	8.96±3.22
**Apical dendritic length (mm**)	3.77±1.24	4.57±2.42	3.78±1.14	5.24±2.51	3.88±1.8	4.52±1.63	4.64±1.25
**Basal dendritic length (mm**)	2.97±1.63	3.93±2.74	2.84±1.33	4.04±2.44	2.88±1.27	4.18±1.35	4.31±2.13
**Total number of nodes**	37.42±23.58	40.88±20.54	35.36±12.07	48.36±24.23	32.11±12.38	47.33±15.57	48.38±16.47
**Max. horizontal extension (μm**)	403.33±118.55	455.24±114.42	417.2±98.1	472.2±104.51	448.3±75.94	393.7±90.29	420.93±77.49
**Max. vertical extension (μm**)	498.12±87.85	521.21±102.88	463.83±94.63	529.58±129.28	447.41±114.17	488.02±81.02	490.45±100.53
**Apical terminal length (μm**)	182.42±48.17	204.83±49.62	162.47±21.5	189.59±42.54	199.68±36.2	151.23±23.3	174.62±36.76
**Basal terminal length (μm**)	142.54±32.43	142.69±30.31	138.7±31.22	150.32±26.09	155.29±32.15	131.6±30.44	148.96±38.42

**Figure 5—figure supplement 1.**
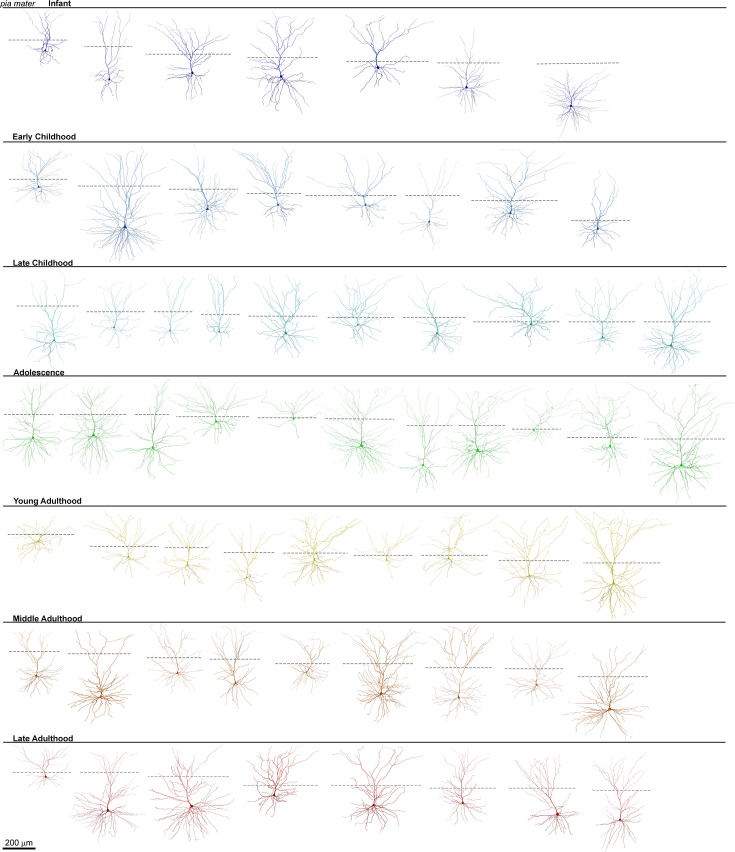
Human cortical L2/3 pyramidal cell dendritic reconstructions. Reconstructions (n=63) of the examined human cortical pyramidal cells, from top to bottom: infant, early childhood, late childhood, adolescence, young adulthood, middle adulthood, and late adulthood age groups. Black lines represent the pia mater, gray dashed lines represent the L1-L2 border.

**Figure 5—figure supplement 2.**
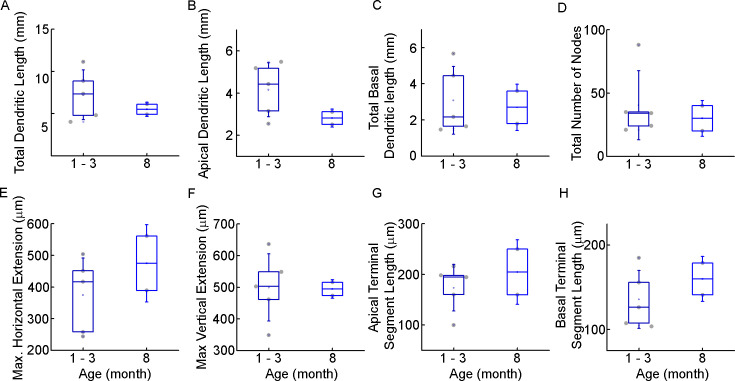
Morphological comparison of the examined infant cells. (**A–H**) Boxplots show morphological features: total (**A**), apical (**B**), total basal (**C**) dendritic length, total number of nodes (**D**), maximal horizontal (**E**), and vertical (**F**) extension, average apical (**G**) and basal (**H**) terminal dendritic segment from infant patient during the first and second half of the first year of life.

**Figure 5—figure supplement 3.**
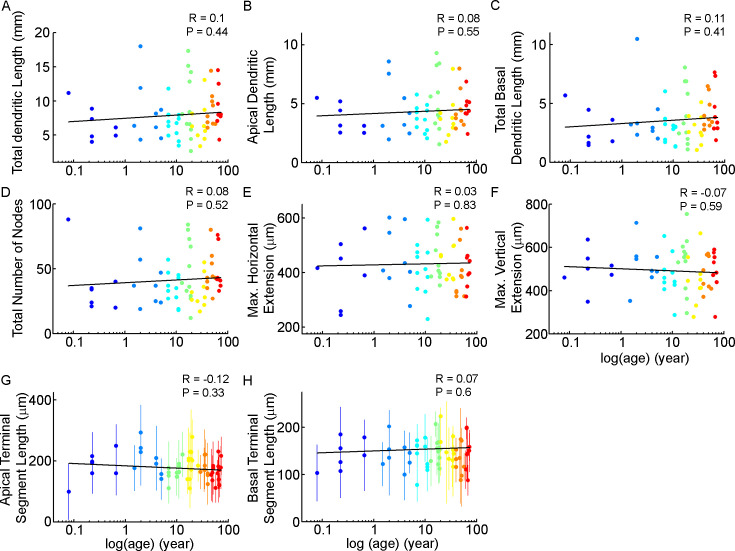
Distribution of morphological features with age. (**A–F**) Scatterplots show the distribution of total (**A**), apical (**B**), total basal dendritic length (**C**), total number of bifurcations (**D**), maximum horizontal extension (**E**), maximum vertical extension of the dendritic arborisation (**F**). The age is represented in years on a logarithmic scale. G-H: Scatterplots show the average apical (**G**) and basal (**H**) terminal segment length of the cells, dots indicate the mean segment length of cells, vertical lines show the standard deviation.

**Figure 6.**
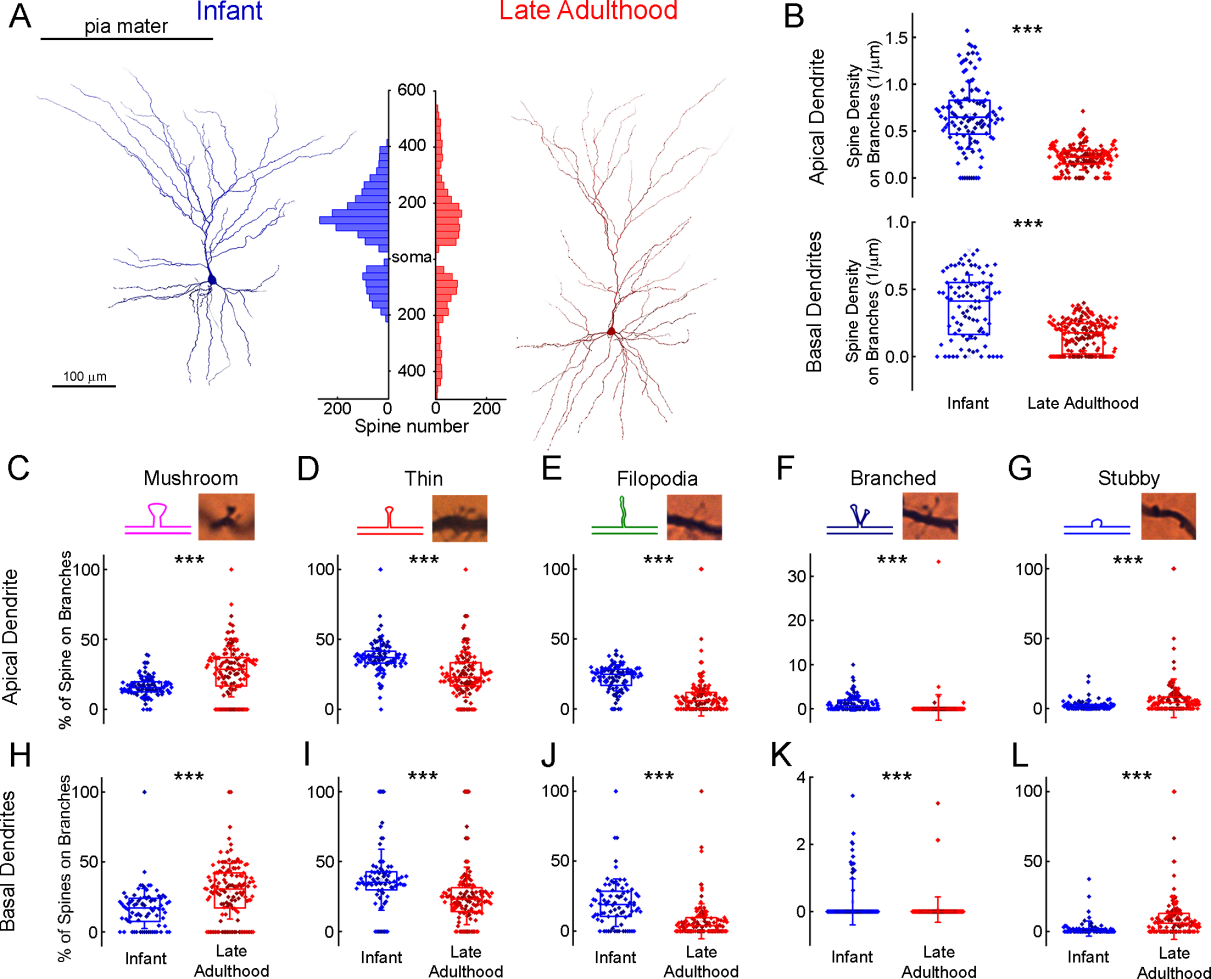
Comparison of dendritic spine densities in pyramidal cells from infant and late adulthood samples. (**A**) Anatomical 3D reconstruction of human L2/3 pyramidal cells from the infant (left), and the late adulthood (right) age groups. The histogram (in the middle) demonstrates the distribution of dendritic spines on the two representative cells according to their distance from the soma (μm) on the apical and basal dendrites. (**B**) Boxplots of the average spine densities on the apical (top), and basal (bottom) dendritic branches from the n=3 infant (blue) and n=3 late adulthood (red) L2/3 pyramidal cells. The symbols are color-coded by the 6 individual cells.(**C–G**) The plots show the distribution of mushroom (**C**), thin (**D**), filopodium (**E**), branched (**F**), and stubby (**G**) dendritic spine types on the apical dendrites of the reconstructed infant (n=3, blue) and late adult (n=3, red) pyramidal cells. Top, schematic illustration and representative images of the examined dendritic spine types. Center, age-dependent distribution of spine types. Asterisks indicate significance (*p<0.05, **p<0.01, ***p<0.001). (**H–L**) Same as C-G but on basal dendrites.

To analyze the distribution of dendritic spines we identified and labeled each spine on n=6 fully 3D-reconstructed cells (Figure 6, Supplementary file 2). We compared the spine density of selected pyramidal cells of two age groups: infant (83 d old, n=3 of one patient, parietal lobe) vs. late adulthood (64.3±2.08 y old, n=3 of 3 patients, frontal, temporal and parietal lobes). The investigated cells are located in L2 (infant: 144.43±45.26 µm, late adulthood: 161.22±66.22 µm). We found that the total spine density was higher (p=7.57 × 10^–40^, Mann-Whitney test) and also the spine density of both apical (p=2.02 × 10^–31^, Mann-Whitney test) and basal (p=3.8 × 10^–12^, Mann-Whitney test) dendrites was higher in the infant than in the late adult group (Figure 6B). To evaluate the age-dependence of spine morphology, we classified the spines into commonly used phenotypes based on their morphological characteristics (Li et al., 2023), specifically distinguishing between mushroom-shaped, thin, filopodial, branched, and stubby spines (Figure 6C–G). Spines with large spine heads were classified as mushroom-shaped, those with small heads as thin, and long protrusions were distinguished as filopodial. Those that did not have peduncles were classified as stubby, spines with two heads emerging from the same spot were called branched spines (Figure 6C–G; Luebke et al., 2015). Only fully visible spines were included in the classification analysis. The composition of spine types varies between the two age groups, mushrooms are present at a higher percentage on apical branches in late adulthood cells (p=4.4 × 10^–9^, Mann-Whitney test), and on the basal processes (p=9.04 × 10^–8^, Mann-Whitney test) (Figure 6C and H). In contrast, thin spines and filopodia are present in significantly higher numbers on the apical (Figure 6D and E) (thin spines: p=7.34 × 10^–14^; filopodia: p=1.11 × 10^–39^, Mann-Whitney test) and basal (thin: p=2.46 × 10^–8^; filopodia: p=2.14 × 10^–12^, Mann-Whitney test) (Figure 6I–J) dendritic branches of the infant. Both apical and basal infant branches had a significantly higher percentage of branched spines (apical: p=1.64 × 10^–11^; basal: p=8.9 × 10^–5^, Mann-Whitney test) (Figure 6F and K), which were present in modest numbers on both. Stubby spines were also more prevalent on the elderly pyramidal cells, either on the apical (p=7.19 × 10^–5^, Mann-Whitney test) or basal (p=6.97 × 10^–9^, Mann-Whitney test) processes (Figure 6G and L). Comparing the spine density of the six individual cells, we found differences across the two age groups similar to those previously mentioned, alongside slight variation within age groups. (Supplementary file 2 Supplementary file 2, Kruskal-Wallis test with post-hoc Dunn test).

**Figure 6—figure supplement 1.**
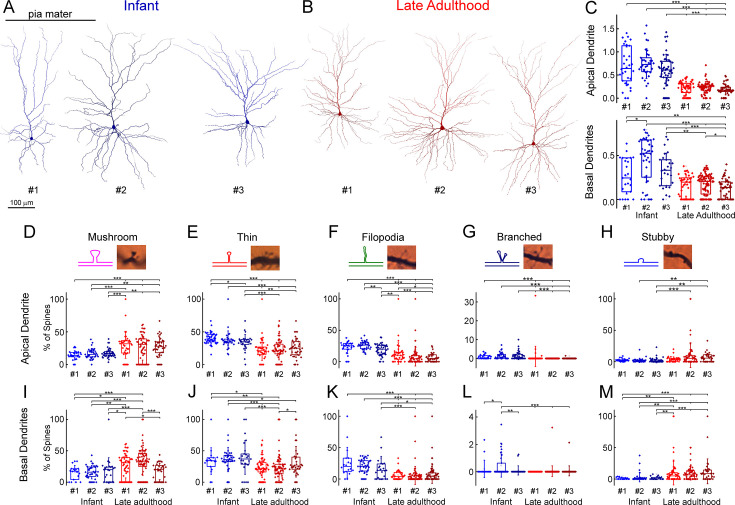
Comparison of dendritic spine densities in pyramidal cells from all samples. (**A**) Anatomical 3D reconstructions of the examined n=3 human L2/3 pyramidal cells from the infant age group. (**B**) Anatomical reconstructions (n=3) from the late adulthood group. (**C**) Boxplots showing the spine density on the six individual cells, the infant pyramidal cell shown with blue, the late adulthood pyramidal cell with red (cells are numbered according to panels **A** and **B**), on the apical (top) and the basal (bottom) dendrites. Asterisks indicate significance (*p<0.05, **p<0.01, ***p<0.001, Kruskal-Wallis test with post-hoc Dunn test). (**D–H**) The plots show the distribution of mushroom (**D**), thin (**E**), filopodium (**F**), branched (**G**), and stubby (**H**) dendritic spine types on the apical dendrites of the reconstructed infant (n=3, blue) and late adult (n=3, red) pyramidal cells. Top, schematic illustration and representative images representation of the examined dendritic spine types. The width of the inserted images is 5 μm. Bottom, spine distributions on the individual cells that were examined. Asterisks indicate significance (*p<0.05, **p<0.01, ***p<0.001, Kruskal-Wallis test with post-hoc Dunn test). (**I–M**) Same as D-H but on basal dendrites.

**Figure 6—figure supplement 2.**
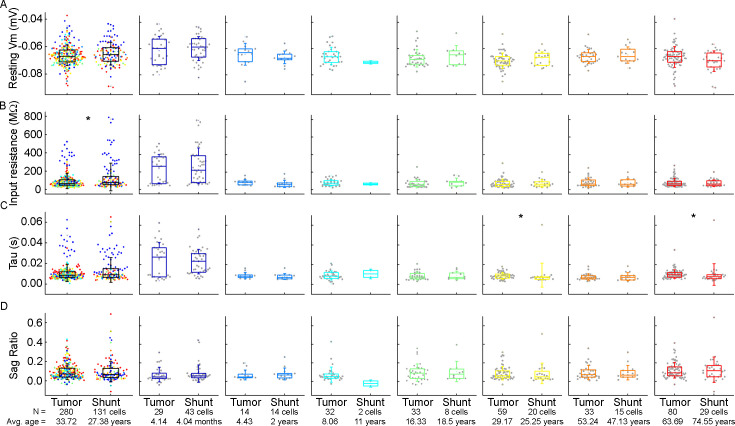
Comparison of subthreshold electrophysiological properties of the examined cells from patients with tumor removal or ventriculoperitoneal (VP) shunt surgical procedures. (**A–D**) Boxplots show resting membrane potential (**A**), input resistance (**B**), tau (**C**), and sag ratio (**D**) from patients with tumor removal (tumor) or VP shunt (shunt) surgical procedures. From left to right: data from all age groups, infant, early childhood, late childhood, adolescence, young adulthood, middle adulthood, and late adulthood groups. Asterisks indicate significance (*p<0.05, Mann-Whitney test).

**Figure 6—figure supplement 3.**
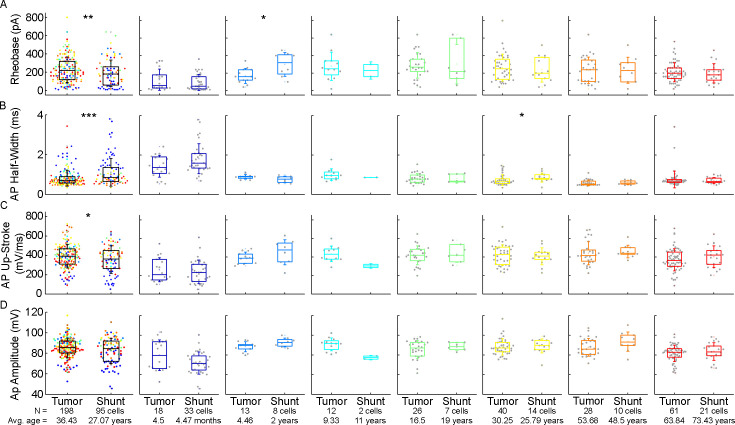
Comparison of action potential properties of the examined cells from patients with tumor removal or ventriculoperitoneal (VP) shunt surgical procedures. (**A–D**) Boxplots show rheobase (**A**), action potential half-width mean (**B**), action potential up-stroke (**C**) and action potential amplitude (**D**) from patients with tumor removal (tumor) or VP shunt (shunt) surgical procedures. From left to right: data from all age groups, infant, early childhood, late childhood, adolescence, young adulthood, middle adulthood, and late adulthood groups. Asterisks indicate significance (*p<0.05, **p<0.01, ***p<0.001, Mann-Whitney test).

**Figure 6—figure supplement 4.**
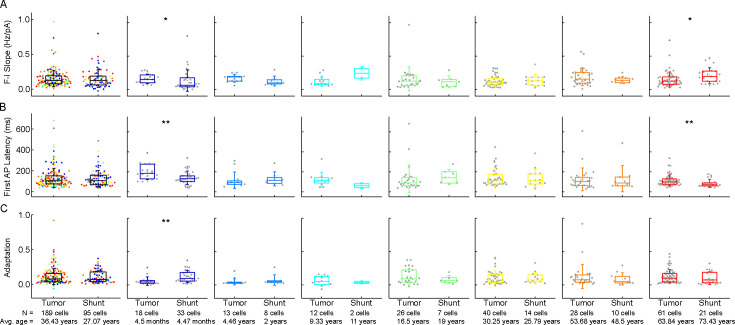
Comparison of firing pattern characteristics of the examined cells from patients with tumor removal or ventriculoperitoneal (VP) shunt surgical procedures. (**A–C**) Boxplots showing: F-I slope (**A**), first action potential (AP) latency (**B**), and adaptation of APs (**C**) from patients with tumor removal (tumor) or VP shunt (shunt) surgical procedures. From left to right: data from all age groups, infant, early childhood, late childhood, adolescence, young adulthood, middle adulthood, and late adulthood groups. Asterisks indicate significance (*p<0.05, **p<0.01, Mann-Whitney test).

**Figure 6—figure supplement 5.**
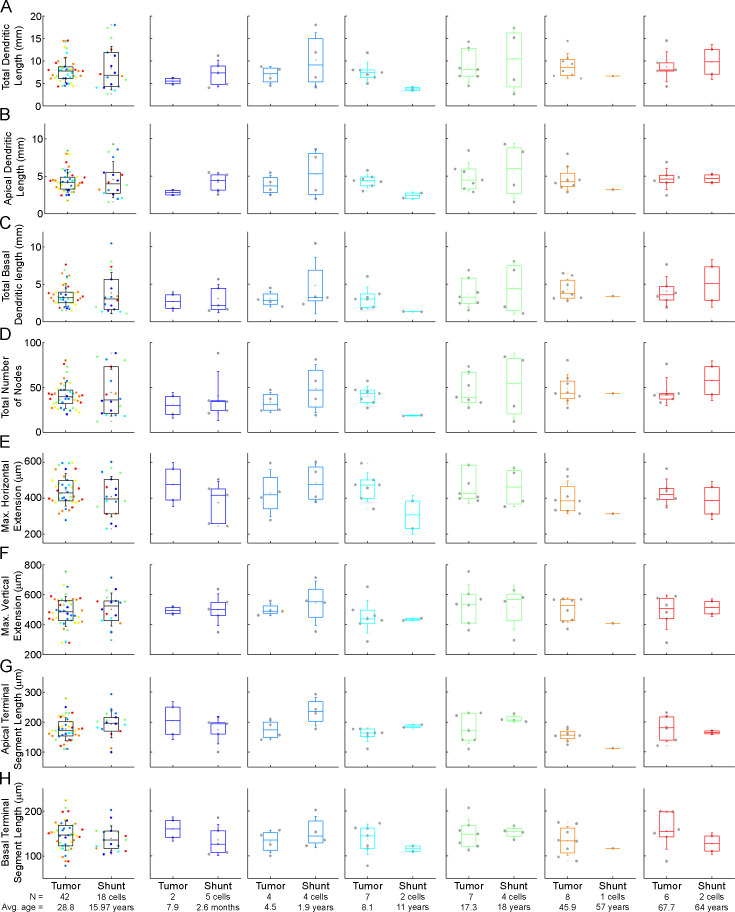
Morphological comparison of the examined cells from patients with tumor removal or ventriculoperitoneal (VP) shunt surgical procedures. (**A–H**) Boxplots showing morphological features: total (**A**), apical (**B**), total basal (**C**), dendritic length, total number of nodes (**D**), maximal horizontal (**E**), and vertical (**F**) extension, average apical (**G**) and basal (**H**) terminal dendritic segment from patients with tumor removal (tumor) or VP shunt (shunt) surgical procedures. From left to right: data from all age groups, infant, early childhood, late childhood, adolescence, middle adulthood, and late adulthood groups. Asterisks indicate significance (*p<0.05, Mann-Whitney test).

**Figure 6—figure supplement 6.**
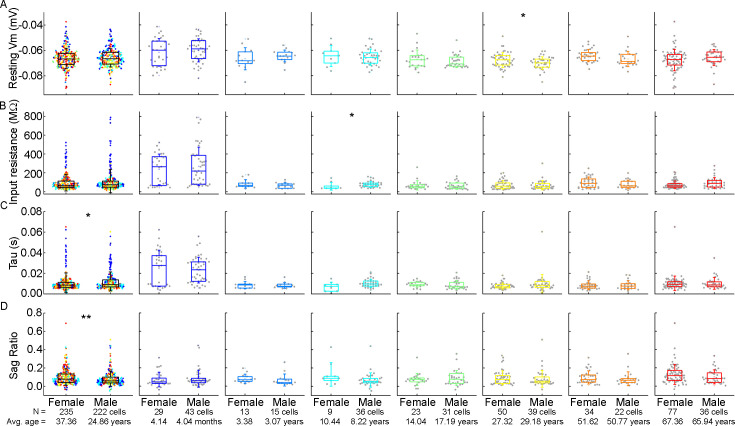
Comparison of subthreshold electrophysiological properties of the examined cells from female and male patients. (**A–D**) Boxplots show resting membrane potential (**A**), input resistance (**B**), tau (**C**), and sag ratio (**D**) from female and male patients. From left to right: data from all age groups, infant, early childhood, late childhood, adolescence, young adulthood, middle adulthood, and late adulthood groups. Asterisks indicate significance (*p<0.05, **p<0.01 Mann-Whitney test or two-sample t-test).

**Figure 6—figure supplement 7.**
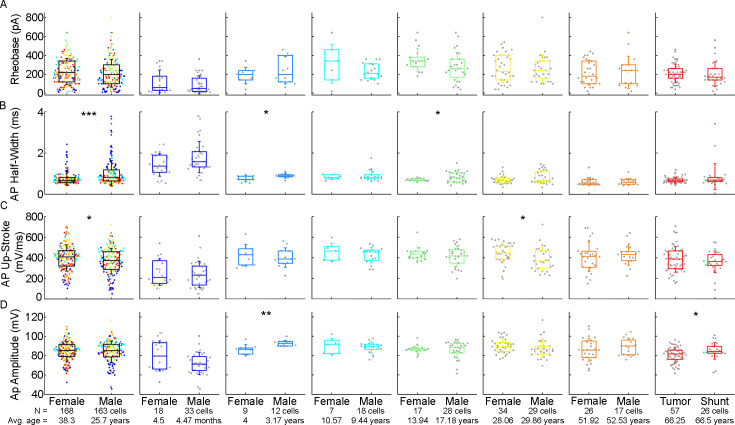
Comparison of action potential properties of the examined cells from female and male patients. (**A–D**) Boxplots show rheobase (**A**), action potential half-width mean (**B**), action potential up-stroke (**C**) and action potential amplitude (**D**) from female and male patients. From left to right: data from all age groups, infant, early childhood, late childhood, adolescence, young adulthood, middle adulthood, and late adulthood groups. Asterisks indicate significance (*p<0.05, **p<0.01, ***p<0.001, Mann-Whitney test or two-sample t-test).

**Figure 6—figure supplement 8.**
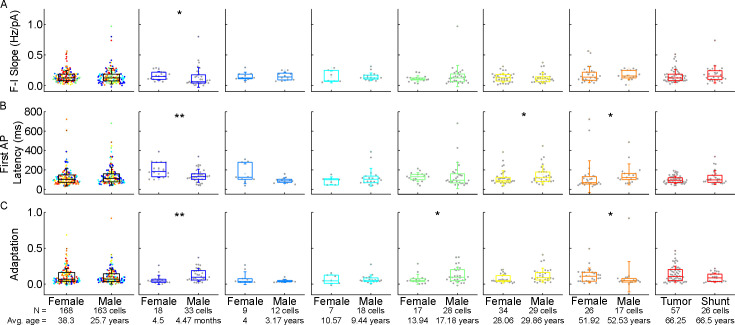
Comparison of firing pattern characteristics of the examined cells from female and male patients. (**A–C**) Boxplots showing: F-I slope (**A**), first action potential (AP) latency (**B**), and adaptation of APs (**C**) from female and male patients. From left to right: data from all age groups, infant, early childhood, late childhood, adolescence, young adulthood, middle adulthood, and late adulthood groups. Asterisks indicate significance (*p<0.05, **p<0.01, Mann-Whitney test or two-sample t-test).

**Figure 6—figure supplement 9.**
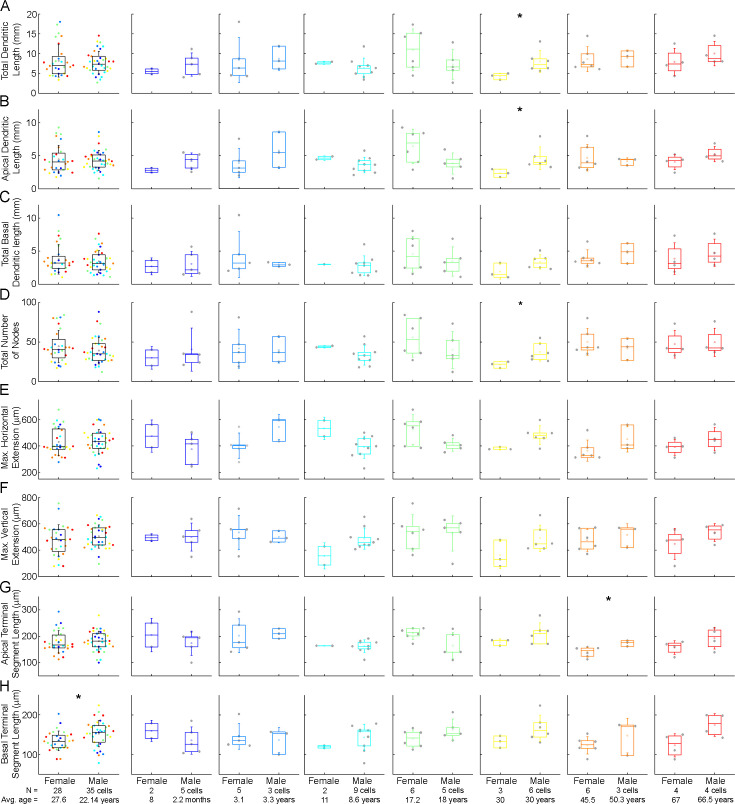
Morphological comparison of the examined cells from female and male patients. (**A–H**) Boxplots showing morphological features: total (**A**), apical (**B**), total basal (**C**), dendritic length, total number of nodes (**D**), maximal horizontal (**E**), and vertical (**F**) extension, average apical (**G**), and basal (**H**) terminal dendritic segment from female and male patients. From left to right: data from all age groups, infant, early childhood, late childhood, adolescence, and late adulthood groups.

Cortical tissue was dissected during neurosurgical procedures for various pathologies, but not to the same extent in the different groups. Specimen collection in adult/elderly patients was mostly for tumor resection, whereas in children hydrocephalus was the most common reason for brain surgery (Figure 1—figure supplement 1). To determine the difference in medical condition could contribute to age-related differences in cellular features, we compared the extracted electrophysiological and morphological features based on the medical condition in the different age groups. It should be noted that all circumstances of surgical steps, tissue dissection, transport (time, media, temperature etc.) and cutting procedure remained the same in the different conditions. When comparing passive electrophysiological properties, we found no significant differences between the different medical conditions in the age groups from infancy to middle adulthood, only time constants were found to be significantly lower in the hydrocephalus patients than in the tumor patients in the young adulthood (p=0.048, Mann-Whitney test) and late adulthood groups (p=0.01, Mann-Whitney test) (Figure 6—figure supplement 2). Comparing the action potential kinetics and firing pattern-related parameters between the different pathology groups we found no overall differences with some sporadic exceptions. For example, the parameters of AP half-width in young adulthood (p=0.01, Mann-Whitney test) and the values of rheobase in early childhood (p=0.04, Mann-Whitney test) differed significantly between the different pathologies (Figure 6—figure supplement 2). Regarding the firing pattern-related properties we found significant changes in the infant F-I slope (p=0.02, Mann-Whitney test), first AP latency (p=0.006, two-sample t-test) and adaptation (p=0.002, Mann-Whitney test) parameters. Also in the late adulthood group the F-I slope (p=0.04, Mann-Whitney test), the first AP latency (p=0.02, Mann-Whitney test) and the adaptation (p=0.047, Mann-Whitney test) were significantly different between the hydrocephalus and tumor patients (Figure 6—figure supplement 4). Further comparison of morphological features revealed no statistical difference between tumor and hydrocephalus groups (Figure 6—figure supplement 5).

To ascertain whether age-related variations in cell properties may be influenced by the gender of the patient, we compared the examined characteristics of the cells by their sex (Figure 6—figure supplements 6 and 7 and 8). Comparing the passive membrane properties of the cells in different age groups we found higher resting membrane potential values in adolescent females than in males (p=0.04, Two-sample t-test) and input resistance was lower in female patients in the late childhood group (p=0.03, Mann-Whitney test) (Figure 6—figure supplement 6). We found differences when comparing the AP characteristics in AP half-with cells from male patient showed higher values in early childhood (p=0.036) and adolescence (p=0.02). AP up-stoke velocity was higher in young adulthood females than in males (p=0.34). AP amplitude was significantly higher in male patients in early childhood (p=0.003) and late adulthood (p=0.035) age groups (Figure 6—figure supplement 7). Under the comparison of the firing pattern features cells from the infancy showed significant differences in F-I slope (p=0.2, Mann-Whitney test), first AP latency (p=0.0096, Two-sample t-test), and adaptation (p=0.002, Mann-Whitney test). The latency of the first AP was higher in cells from male patient in the young (p=0.03, Mann-Whitney test) and middle (p=0.018, Mann-Whitney test) adulthood cells. The adaptation of the APs in males was higher during adolescence (p=0.027, Mann-Whitney test) but lower in middle adulthood (p=0.034, Mann-Whitney test) (Figure 6—figure supplement 8). Regarding the morphological property of the reconstructed cell we found differences in the young adulthood age group between cells from male and female patients in total (p=0.03, Mann-Whitney test) and apical (p=0.03, Mann-Whitney test) dendritic length and the total number of bifurcations (p=0.03, Mann-Whitney test), in the middle adulthood group we found significant difference in the average apical terminal length across males females(p=0.03, Mann-Whitney test) (Figure 6—figure supplement 9).

## Discussion

In this study, using the whole-cell patch-clamp technique and 3D reconstructions, we have studied the differences of human cortical L2/3 pyramidal cells at various stages of life. We found that during the lifespan the most significant changes take place early in life during the first year in most of the biophysiological characteristics of human pyramidal cells (see Figure 2—figure supplement 2). We recorded from n=457 human cortical excitatory pyramidal cells from the supragranular layer from birth to 85 y. Most differences in sub- and suprathreshold features were found in the youngest age groups. There were particular differences in resting membrane potential, input resistance, time constant, rheobasic current, AP halfwidth, and AP upstroke velocity. Other age groups that differed the most from other age groups were the oldest (60–85 y) with modest differences in sag ratios. In our morphological analysis, we found no significant changes in the overall apical and basal dendritic dimensions across different ages. When evaluating the number of spines in two anatomical reconstructions (from the infant and late adulthood groups) we found a higher overall spine density in the infant than in the elder sample consisting of mainly branched and thin spines and filopodia. In contrast, stubby and mushroom-shaped spines were more prevalent in the older pyramidal cell.

The nervous system experiences a multitude of plastic changes throughout an individual’s lifetime. These changes extend from the early stages of development and continue through the gradual degenerative processes that come with old age. Although precise intrinsic cellular modifications linked to aging remain somewhat elusive, there is an observable morphological transformation during early development. As the nervous system matures, dendritic branching (Kasper et al., 1994; McAllister, 2000) and cell body size increases (Zhang, 2004). Also, learning and experience increase the extent of dendritic branching (Holloway, 1966; Greenough and Volkmar, 1973) and dendritic spines (Globus et al., 1973). An extensive body of research has shown that synaptic plasticity induces formation of new dendritic spines and the enlargement of existing spines (Kastellakis et al., 2023). These modifications may affect the input-output relation of a neuron as model studies suggest that dendritic geometry influences electrical and firing properties (Mainen and Sejnowski, 1996; van Elburg and van Ooyen, 2010; Eyal et al., 2014; Vetter et al., 2001). Furthermore, in parallel to the changes in morphological complexity, there is ample experimental evidence that the maturation of ion channels and the change in increasing channel density in the neuronal membrane also play a role in influencing the electrical properties of neurons (Huguenard et al., 1988; Connors, 1994). These findings point to a close causal relationship between structure and electrophysiological properties at cellular level.

### Lifespan changes in electrophysiological properties

The intrinsic electrophysiological properties of a neuron are determined by several factors: the resistivity of the cytoplasm and membrane, the membrane capacitance, and the shape of the soma and dendrites. The basic passive parameters we recorded, such as the resting membrane potential, the input resistance, and the membrane time constant show a dramatical change in the first year. This change is consistent with data found in previous studies on neocortical pyramidal cells from rodent models (Kasper et al., 1994; Kroon et al., 2019; Picken Bahrey and Moody, 2003; Zhang, 2004; Zhu, 2000; McCormick and Prince, 1987) and on xenotransplanted human cortical neurons (Linaro et al., 2019). We found a progressive shift of the resting membrane potential from –60 to –68 mV in the first part of the lifespan (<40 y), which could be due to the age-dependent change in ion channel composition (Picken Bahrey and Moody, 2003). The input resistance and time constant showed a sudden and sharp decrease between the first and second age groups, indicating the most dramatic changes in the infant’s brain followed by a generalized and slower decrease. As pyramidal cells mature, the size and volume of the cell body increase (Petanjek et al., 2019; Zhu, 2000) which correlates with the input resistance, meaning that a small membrane area has a higher input resistance (Luhmann et al., 2000). In addition to the increase in cell surface area, the increase in the expression of certain potassium leak channels, e.g., from the two-pore domain potassium channel (KCNK) family, can also influence the input resistance during cell maturation (Aller and Wisden, 2008; Goldstein et al., 2001). Similar reasons can lead to a decrease in the membrane time constant. The membrane time constant is the product of the specific input resistance and the specific membrane capacitance. Assuming constant membrane capacitance (~1 pF/cm^2^, Oláh et al., 2024, Beaulieu-Laroche et al., 2018), the decrease is likely also due to a change in specific membrane resistance. The result of developmental changes in membrane passive parameters during early development improves spike timing precision and temporal responsiveness in the cortical microcircuit (Oswald and Reyes, 2011; Doischer et al., 2008). However, in our human samples at later ages (>40 y) that we associate with the aging brain, the previous trends of changes in intrinsic characteristics are reversed. Input resistance and time constant increase, which may be associated with the decline in KCNK expression in the aging brain (Erraji-Benchekroun et al., 2005). These membrane parameters are also generally higher in studies in rhesus monkeys (Moore et al., 2023), except for the resting membrane potential, which is not different from that of young cortical L2/3 (Chang et al., 2005) or L5 pyramidal cells (Luebke and Chang, 2007) in the brain. However, in our samples, we found a positive shift in the resting potential between the middle- and old-age groups by a few millivolts. It should be noted that this discrepancy may be due to the fact that the age groups in our work are not the same as the works mentioned.

Changes in resting membrane potential and input resistance will add up to produce a rising and then falling curve of the averaged rheobasic current, with the value three times higher in the adolescent group than in the youngest group and 1.5 times higher than in late adulthood. Our results on increased rheobase during early life are consistent with previous studies in rodent cortical L3 and 5 pyramidal cells (Kroon et al., 2019; Perez-García et al., 2021; Popescu et al., 2021) and with findings regarding aging in rhesus monkey cortical neurons from rhesus monkeys (Moore et al., 2023).

When we assessed the voltage sag ratio with age we found a consistent increase that peaked in late adulthood. The voltage sag that occurs during membrane hyperpolarization is the result of activation of the hyperpolarization-activated cyclic nucleotide-gated (HCN) current, also known as the h-current. Similar to our findings the age-dependent increase in sag deflection has been observed in human cortical pyramidal cells (Guet-McCreight et al., 2023; Kalmbach et al., 2018) which is consistent with studies by Wang et al., 2007; Wang et al., 2011 that have linked inhibited HCN activation to working memory deficits in the elderly.

The electrical output of neurons is driven by the composition of neuronal molecules and ion channels whose expressions and distributions change most dynamically in two periods, early development, and the aging brain. This dynamic essentially produced an inverted U-shaped curve in the distribution of the active electrical properties over the lifespan in our dataset, an increase in the first year, a relatively stable middle phase, and then a return in older age groups. The origin of these changes is mainly due to changes in the distribution of sodium and potassium channels. During development, there is evidence that the number of voltage-gated Na^+^ channels increases in rodents (Huguenard et al., 1988; Picken Bahrey and Moody, 2003) and in humans (Erraji-Benchekroun et al., 2005), which may accelerate the kinetics of the action potential (up-stroke) and, similar to our results, increase the amplitude of the action potential (Perez-García et al., 2021; Kroon et al., 2019; Zhang, 2004; McCormick and Prince, 1987; Etherington and Williams, 2011). During aging, however, regulation is disrupted by the change in channel expression, which can lead to altered conduction rate of ionic currents. In brain senescence, the amplitude of the action potential and a slowing of the action potential Luebke and Chang, 2007 have been shown to be similar to our data, which may be caused by the decreased expression of voltage-gated Na^+^ channels (Erraji-Benchekroun et al., 2005).

We examined the firing patterns of pyramidal cells at different ages by comparing the F-I slope, and we found that the patterns are fairly conserved across ages. In rodent developmental studies, a decrease in F-I slope was found only in the first 2 wk after birth (Zhang, 2004; McCormick and Prince, 1987), which time window is not represented in our data. The latency of the first spike is influenced by the time-constant and specific ion channels, like the transient (or A-type) K^+^ current and the transient Ca^2+^ current (or T-type) (Molineux et al., 2005). The same ion channels are involved in burst firing at the onset of stimulation. Note that in our sample only ~10% of pyramidal cells showed burst firing at the beginning of stimulation, but it is known that burst firing is more frequent from layer 3 onwards at depth (Berg et al., 2021). The presence of A-current has been found in immature pyramidal neurons from sensorimotor areas in rodent studies (McCormick and Prince, 1987), and the density of T-type currents also remains unchanged during the development of the visual cortex (Horibe et al., 2014). It has been shown that the expression of the T-type calcium channel family (CACNG3) decreases during aging (Erraji-Benchekroun et al., 2005). However, it must be mentioned that the differences in the expression of all subtypes of the Kv4 (A-type) and Ca_V_3.x (T-type) channel families during brain development and aging have not been systematically described.

### Properties of dendritic trees and spine distribution with age

Transcriptomic analyses of the human neocortex show relatively stable expression patterns of genes for dendritic and synaptic development after about 1 y of age (Kang et al., 2011). In early postmortem studies, human pyramidal neurons were studied for morphological changes as a function of age. In these studies, using Golgi staining methods, it was found that the dendritic trees of infant layer 2/3 pyramidal cells are well developed (Mrzljak et al., 1990). In the human prefrontal cortex after birth, the dendritic tree of layer 3 pyramidal neurons reaches its structurally mature form after about 3 m (Petanjek et al., 2008). Thereafter, it is assumed that a smaller increase in dendritic length reaches its final form between about 7.5–12 m (Koenderink and Uylings, 1995; Petanjek et al., 2008). In our study, we observed stable patterns of apical and basal dendrite lengths and dendritic tree complexity across the lifespan. Even between the early postnatal groups (infancy vs. early childhood), no significant difference in dendritic tree size was observed. This can be explained by the influence of various factors, such as the large difference between individual subjects or the subtype-specific dendritic morphology of pyramidal cells (Berg et al., 2021) or different maturation curves of neurons from different brain regions (Elston and Fujita, 2014) or cortical layers (Petanjek et al., 2008).

Neurons exhibit a phase of synaptogenesis overproducing synapses lasting months or years in the human cortex (Huttenlocher, 1979; Huttenlocher et al., 1982; Mrzljak et al., 1990; Petanjek et al., 2011), followed by dendritic spine/synapse pruning that is reported to last more than a decade in the cortex (Petanjek et al., 2011; Jacobs et al., 1997; Mavroudis et al., 2015; Benavides-Piccione et al., 2013; Coskren et al., 2015). Our results from comparing two groups: infant and late adult pyramidal cells also show that the overall number of dendritic spines decreases with increasing age. When evaluating the distribution of spine shapes in young and old pyramidal cells, we found spines with larger heads (mushroom shape) in a greater number in the dendrite of old pyramidal cells, which are considered as mature synapses. The size of the head is an indicator of the size of the postsynaptic density, the number of glutamate receptors, and the strength of the synapse (Bourne and Harris, 2008; Matsuzaki et al., 2001; Nusser et al., 1998). In the developing nervous system, however, the filopodia, thin dendritic protrusions without a head, are the most characteristic type of spines. The density of filopodial spines was higher on the dendrites of infant pyramidal cells. In the older cells, we still found filopodia, which are the silent precursor of active synapses (Vardalaki et al., 2022). The change in the ratio of subtypes from young to old age is well established and is associated with the basis for changes in cognitive function during aging (Luebke et al., 2015; Dumitriu et al., 2010; Jacobs et al., 1997).

The complexity of human brain activity and cognitive abilities increases with development and decreases with aging, resulting in an inverted U-shaped curve across the lifespan (Craik and Bialystok, 2006). Cognitive functions depends on many age-dependent factors, such as the density and specificity of synaptic connections formed by synaptic pruning and plasticity, or the degree of myelination and white matter maturation and there is also an age-related modulation of neurotransmitters, hormones, ion transporters, and receptors (Luna et al., 2015). Here, we have shown that pyramidal cells become less excitable and temporarily more precise during development by changing their intrinsic functional properties and with aging these changes occur somewhat in opposite directions making the intrinsic parameters change symmetrically. In addition, some of the changes are asymmetrical either occur with development or with aging, such as the resting membrane potential which mostly changes in young ages or the ratio of sag, which is shifted most in old age. These changes in the intrinsic properties of cells during the first and last stages of life also contribute to the input-output functions of a neuron and ultimately to the age-related development of cognitive abilities.

## Materials and methods

### Slice preparation

Experiments were performed according to the Declaration of Helsinki with the approval of the University of Szeged Ethical Committee and Regional Human Investigation Review Board (ref. 75/2014). Prior to surgery, the patients provided written consent for all tissue material. We used human cortical tissue adjacent to the pathological lesion that had to be surgically removed from patients (n=64 female n=45 male) as part of the treatment for tumors, hydrocephalus, apoplexy, cysts, and arteriovenous malformation. Anesthesia was induced with intravenous midazolam and fentanyl (0.03 mg/kg, 1–2 µg/kg, respectively). A bolus dose of propofol (1–2 mg/kg) was administered intravenously. The patients received 0.5 mg/kg rocuronium to facilitate endotracheal intubation. The trachea was intubated, and the patient was ventilated with a mixture of O_2_-N_2_O at a ratio of 1:2. Anesthesia was maintained with sevoflurane at a care volume of 1.2–1.5. During the surgical procedure tissue blocks were removed from parietal (n=22), temporal (n=35), frontal (n=42), and occipital (n=10) regions, the resected tissue blocks were immediately immersed in ice-cold solution. Slices were cut perpendicular to the pia mater at a thickness of 320 µm with a vibrating blade microtome (Microm HM 650 V) in ice-cold solution (in mM) 75 sucrose, 84 NaCl, 2.5 KCl, 1 NaH_2_PO_4_, 25 NaHCO_3_, 0.5 CaCl_2_, 4 MgSO_4_, 25 D(+)-glucose, saturated with 95% O2 and 5% CO2. The slices were incubated in the same solution for 30 min at 36 °C following that the solution was changed to (in mM) 130 NaCl, 3.5 KCl, 1 NaH_2_PO_4_, 24 NaHCO_3_, 1 CaCl_2_, 3 MgSO_4_, 10 D(+)-glucose, saturated with 95% O_2_ and 5% CO_2_, the slices were kept in it until use.

### In vitro electrophysiological recordings

Somatic whole-cell current-clamp recordings were obtained at ~36 °C in solution containing (in mM) 130 NaCl, 3.5 KCl, 1 NaH_2_PO_4_, 24 NaHCO_3_, 3 CaCl_2_, 1.5 MgSO_4_, 10 D(+)-glucose, from layer 2/3 pyramidal cells visualized by infrared differential interference contrast (DIC) video microscopy equipped with micromanipulators (Luigs and Neumann, 652 Ratingen, Germany) and HEKA EPC 9&10 patch clamp amplifier (HEKA Elektronik GmbH, Lambrecht, Germany). Micropipettes (3–5 MΩ) were filled with intracellular solution containing (in mM) 126 potassium-gluconate, 4 KCl, 4 ATP-Mg, 0.3 GTP-Na_2_, 10 HEPES, 10 phosphocreatine, and 8 biocytin (pH 7.20; 300 mOsm). After whole-cell configuration was obtained stepwise currents were injected to measure the evoked sub- and suprathreshold membrane potential properties. For the analysis of the electrophysiological recordings n=457 recordings with a series resistance (Rs) of 24.93±11.18 MΩ (max: 63.77 MΩ) were used. For the analysis of fast parameters related to the action potential (AP half-width, AP upstroke velocity, AP amplitude, and rheobase), higher quality requirements were set and cells with Rs >30 MΩ were excluded. This reduced the data set to n=331 cells with Rs 19.42±6.2 MΩ.

### Data analysis

Electrophysiological features (Table 4) were measured from voltage responses elicited by 800 ms long current steps increasing by 20 pA from –100 pA. We analyzed the electrophysiological data with Fitmaster software (HEKA Elektronik GmbH, Lambrecht), and custom MATLAB (The Math Works, Inc) scripts.

**Table 4.** Examined electrophysiological properties.

Resting Vm	The membrane potential of the neuron, measured directly after attaining the whole-cell configuration with no current (if a holding current was used during the recording we compensated the resting membrane potential with the injected current).
Input resistance	To calculate input resistance the mean of all hyperpolarizing current produced voltage steps were used.
Tau	To calculate time constant the mean of all hyperpolarizing current produced voltage steps were used, measured between 0–63%.
Sag ratio	The ratio of the maximal deflection and the steady-state membrane potential during a –100 pA current step.
Rheobase	The minimal current step that elicited the first spike.
AP half-width	The width of the AP at half amplitude.
AP up-stroke	The mean of all the maximum values of dV/dt between the action potential onset and the action potential peak from each elicited APs of the cell.
AP amplitude	Average amplitude of all APs, from threshold to peak.
F-I slope	The slope of the line fitted to the data of the AP firing frequency versus stimulus intensity.
First AP latency	The duration from the start of the stimulus until the first AP under the rheobasic current step.
Adaptation	The average adaptation of the interspike interval between consecutive APs.
Rebound	The difference between the steady-state membrane potential and the maximum deflection after a hyperpolarizing current step.
Rebound-Sag ratio	The ratio of the rebound and sag amplitudes.
Avg. AP number	Average number of elicited APs per sweep.
AP threshold	Mean of all the voltage values at AP threshold.
Ap rise time	Mean time between the threshold and the peak of all APs.
AHP amplitude	Average amplitude of all afterhyperpolarization.
AHP length	Average duration of 0 (AHP minimum) to 90% of all the AHP.
Voltage at max.dV/dt	Average voltage value at the maximum of the AP dv/dt over all APs
Velocity at min. dV/dt	Average velocity value at the minimum of the AP dv/dt from all APs
Voltage at min. dV/dt	Average voltage value at the minimum of the AP dv/dt from all APs
AP peak	Average of AP maximum voltages
ISI mean	Average interspike interval from all sweeps containing at least three APs
AP amplitude accommodation	The difference of the first and last AP amplitude in a sweep.
AP half-width accommodation	The difference of the first and last AP half-width in a sweep.
AP threshold accommodation	The difference of the first and last AP threshold in a sweep.
Average ISI	The mean value of interspike intervals in a sweep.
AP amplitude adaptation	The average adaptation of the AP amplitude between consecutive APs.
AP half-width adaptation	The average adaptation of the AP half-width between consecutive APs.
AP threshold adaptation	The average adaptation of the AP threshold between consecutive APs.
AHP area	Integral of the AHP from the minimum value to 90%, using trapezoidal method.
ADP amplitude	The amplitude of the afterdepolarization, the average difference between the minimum value of the AHP and the threshold of the next AP.

Analysis of morphological features was made by NeuroExplorer software (MBF Bioscience, Williston, VT, USA) and Origin 9 (OriginLab, Northampton, MA).

### Statistics

We used custom-written R scripts (R 4.1.2) and the gamm function from the mgcv R package (mgcv 1.8.38). Data presented as the mean ± s.d. Normality was tested with the Lilliefors test, for statistical analysis, ANOVA with posthoc Bonferroni test, Kurskal-Wallis with posthoc Dunn test, for pairwise comparison two-sample t-test or Mann-Whitney test was used. Differences were accepted as significant if p<0.05. The data are shown on boxplots, boxes indicate 25^th^, 50^th^ (median), and 75^th^ percentiles, rectangle represents the mean value, and whiskers indicate s.d.

### Histology and reconstruction

Slices were fixed in a fixative of 4% paraformaldehyde, 15% picric acid, and 1.25% glutaraldehyde in 0.1 M phosphate (PB, pH = 7.4) for at least 12 hr after electrophysiological recording. After multiple washes in 0.1 M PB, slices were cryoprotected in 10% then 20% sucrose solution in 0.1 M PB. The slices were frozen in liquid nitrogen and then thawed in PB. Slices were embedded in 10% gelatin and further sectioned into 70 µm thick sections. Sections were incubated in a solution containing conjugated avidin-biotin horseradish peroxidase (ABC; 1:100; Vector Labs) in Tris-buffered saline (TBS, pH = 7.4) overnight at 4 °C. The enzyme reaction became visible by using 0.05% 3'3-diaminobenzidine tetrahydrochloride as a chromogen and 0.01% H_2_O_2_ as an oxidant. Sections were post-fixed with 1% OsO_4_ in 0.1 M PB. Following several washes with distilled water, sections were stained in 1% uranyl acetate, and dehydrated in an ascending series of ethanol. The sections were infiltrated with epoxy resin (Durcupan, Sigma-Aldrich) overnight and embedded on glass slides. After the electrophysiologically recorded cells have been visualized by DAB staining, 3D light microscopic reconstructions were carried out using the Neurolucida system (MBF Bioscience, Williston, VT, USA) with a 100x objective. The length of terminal segments was measured between the terminal tip of the dendrites and the last branching point before the terminal tip. Dendritic spine density on the dendritic branches was calculated as spine/μm between two bifurcations. The percentage of spines on branches was calculated as the percentage of the given spine type from all the types of spines on that branch.

## Data Availability

Source data files have been deposited in Dandiarchive.org under the accession ID: 001281. The following dataset was generated: PálB IldikóS MartinT Éva AdriennC GáborM GáborT 2025Electrophysiology and Morphology of Human Cortical Supragranular Pyramidal Cells in a Wide Age RangeDANDI00128110.7554/eLife.100390PMC1195275140152903

## Additional files

Supplementary file 1. Patients’ data for all subjects used in this study.

Supplementary file 2. Number of dendritic spines of the examined pyramidal cells.

MDAR checklist

## References

[bib1] Aller MI, Wisden W (2008). Changes in expression of some two-pore domain potassium channel genes (KCNK) in selected brain regions of developing mice. Neuroscience.

[bib2] Beaulieu-Laroche L, Toloza EHS, van der Goes MS, Lafourcade M, Barnagian D, Williams ZM, Eskandar EN, Frosch MP, Cash SS, Harnett MT (2018). Enhanced dendritic compartmentalization in human cortical neurons. Cell.

[bib3] Beck D, de Lange AMG, Maximov II, Richard G, Andreassen OA, Nordvik JE, Westlye LT (2021). White matter microstructure across the adult lifespan: a mixed longitudinal and cross-sectional study using advanced diffusion models and brain-age prediction. NeuroImage.

[bib4] Benavides-Piccione R, Fernaud-Espinosa I, Robles V, Yuste R, DeFelipe J (2013). Age-based comparison of human dendritic spine structure using complete three-dimensional reconstructions. Cerebral Cortex.

[bib5] Berg J, Sorensen SA, Ting JT, Miller JA, Chartrand T, Buchin A, Bakken TE, Budzillo A, Dee N, Ding S-L, Gouwens NW, Hodge RD, Kalmbach B, Lee C, Lee BR, Alfiler L, Baker K, Barkan E, Beller A, Berry K, Bertagnolli D, Bickley K, Bomben J, Braun T, Brouner K, Casper T, Chong P, Crichton K, Dalley R, de Frates R, Desta T, Lee SD, D’Orazi F, Dotson N, Egdorf T, Enstrom R, Farrell C, Feng D, Fong O, Furdan S, Galakhova AA, Gamlin C, Gary A, Glandon A, Goldy J, Gorham M, Goriounova NA, Gratiy S, Graybuck L, Gu H, Hadley K, Hansen N, Heistek TS, Henry AM, Heyer DB, Hill D, Hill C, Hupp M, Jarsky T, Kebede S, Keene L, Kim L, Kim M-H, Kroll M, Latimer C, Levi BP, Link KE, Mallory M, Mann R, Marshall D, Maxwell M, McGraw M, McMillen D, Melief E, Mertens EJ, Mezei L, Mihut N, Mok S, Molnar G, Mukora A, Ng L, Ngo K, Nicovich PR, Nyhus J, Olah G, Oldre A, Omstead V, Ozsvar A, Park D, Peng H, Pham T, Pom CA, Potekhina L, Rajanbabu R, Ransford S, Reid D, Rimorin C, Ruiz A, Sandman D, Sulc J, Sunkin SM, Szafer A, Szemenyei V, Thomsen ER, Tieu M, Torkelson A, Trinh J, Tung H, Wakeman W, Waleboer F, Ward K, Wilbers R, Williams G, Yao Z, Yoon J-G, Anastassiou C, Arkhipov A, Barzo P, Bernard A, Cobbs C, de Witt Hamer PC, Ellenbogen RG, Esposito L, Ferreira M, Gwinn RP, Hawrylycz MJ, Hof PR, Idema S, Jones AR, Keene CD, Ko AL, Murphy GJ, Ng L, Ojemann JG, Patel AP, Phillips JW, Silbergeld DL, Smith K, Tasic B, Yuste R, Segev I, de Kock CPJ, Mansvelder HD, Tamas G, Zeng H, Koch C, Lein ES (2021). Human neocortical expansion involves glutamatergic neuron diversification. Nature.

[bib6] Bethlehem RAI, Seidlitz J, White SR, Vogel JW, Anderson KM, Adamson C, Adler S, Alexopoulos GS, Anagnostou E, Areces-Gonzalez A, Astle DE, Auyeung B, Ayub M, Bae J, Ball G, Baron-Cohen S, Beare R, Bedford SA, Benegal V, Beyer F, Blangero J, Blesa Cábez M, Boardman JP, Borzage M, Bosch-Bayard JF, Bourke N, Calhoun VD, Chakravarty MM, Chen C, Chertavian C, Chetelat G, Chong YS, Cole JH, Corvin A, Costantino M, Courchesne E, Crivello F, Cropley VL, Crosbie J, Crossley N, Delarue M, Delorme R, Desrivieres S, Devenyi GA, Di Biase MA, Dolan R, Donald KA, Donohoe G, Dunlop K, Edwards AD, Elison JT, Ellis CT, Elman JA, Eyler L, Fair DA, Feczko E, Fletcher PC, Fonagy P, Franz CE, Galan-Garcia L, Gholipour A, Giedd J, Gilmore JH, Glahn DC, Goodyer IM, Grant PE, Groenewold NA, Gunning FM, Gur RE, Gur RC, Hammill CF, Hansson O, Hedden T, Heinz A, Henson RN, Heuer K, Hoare J, Holla B, Holmes AJ, Holt R, Huang H, Im K, Ipser J, Jack CR, Jackowski AP, Jia T, Johnson KA, Jones PB, Jones DT, Kahn RS, Karlsson H, Karlsson L, Kawashima R, Kelley EA, Kern S, Kim KW, Kitzbichler MG, Kremen WS, Lalonde F, Landeau B, Lee S, Lerch J, Lewis JD, Li J, Liao W, Liston C, Lombardo MV, Lv J, Lynch C, Mallard TT, Marcelis M, Markello RD, Mathias SR, Mazoyer B, McGuire P, Meaney MJ, Mechelli A, Medic N, Misic B, Morgan SE, Mothersill D, Nigg J, Ong MQW, Ortinau C, Ossenkoppele R, Ouyang M, Palaniyappan L, Paly L, Pan PM, Pantelis C, Park MM, Paus T, Pausova Z, Paz-Linares D, Pichet Binette A, Pierce K, Qian X, Qiu J, Qiu A, Raznahan A, Rittman T, Rodrigue A, Rollins CK, Romero-Garcia R, Ronan L, Rosenberg MD, Rowitch DH, Salum GA, Satterthwaite TD, Schaare HL, Schachar RJ, Schultz AP, Schumann G, Schöll M, Sharp D, Shinohara RT, Skoog I, Smyser CD, Sperling RA, Stein DJ, Stolicyn A, Suckling J, Sullivan G, Taki Y, Thyreau B, Toro R, Traut N, Tsvetanov KA, Turk-Browne NB, Tuulari JJ, Tzourio C, Vachon-Presseau É, Valdes-Sosa MJ, Valdes-Sosa PA, Valk SL, van Amelsvoort T, Vandekar SN, Vasung L, Victoria LW, Villeneuve S, Villringer A, Vértes PE, Wagstyl K, Wang YS, Warfield SK, Warrier V, Westman E, Westwater ML, Whalley HC, Witte AV, Yang N, Yeo B, Yun H, Zalesky A, Zar HJ, Zettergren A, Zhou JH, Ziauddeen H, Zugman A, Zuo XN, Bullmore ET, Alexander-Bloch AF, 3R-BRAIN, AIBL, Alzheimer’s Disease Neuroimaging Initiative, Alzheimer’s Disease Repository Without Borders Investigators, CALM Team, Cam-CAN, CCNP, COBRE, cVEDA, ENIGMA Developmental Brain Age Working Group, Developing Human Connectome Project, FinnBrain, Harvard Aging Brain Study, IMAGEN, KNE96, Mayo Clinic Study of Aging, NSPN, POND, PREVENT-AD Research Group, VETSA (2022). Brain charts for the human lifespan. Nature.

[bib7] Bourne JN, Harris KM (2008). Balancing structure and function at hippocampal dendritic spines. Annual Review of Neuroscience.

[bib8] Chang YM, Rosene DL, Killiany RJ, Mangiamele LA, Luebke JI (2005). Increased action potential firing rates of layer 2/3 pyramidal cells in the prefrontal cortex are significantly related to cognitive performance in aged monkeys. Cerebral Cortex.

[bib9] Connors BW (1994). Intrinsic neuronal physiology and the functions, dysfunctions and development of neocortex. Progress in Brain Research.

[bib10] Coskren PJ, Luebke JI, Kabaso D, Wearne SL, Yadav A, Rumbell T, Hof PR, Weaver CM (2015). Functional consequences of age-related morphologic changes to pyramidal neurons of the rhesus monkey prefrontal cortex. Journal of Computational Neuroscience.

[bib11] Craik FIM, Bialystok E (2006). Cognition through the lifespan: mechanisms of change. Trends in Cognitive Sciences.

[bib12] Doischer D, Hosp JA, Yanagawa Y, Obata K, Jonas P, Vida I, Bartos M (2008). Postnatal differentiation of basket cells from slow to fast signaling devices. The Journal of Neuroscience.

[bib13] Dumitriu D, Hao J, Hara Y, Kaufmann J, Janssen WGM, Lou W, Rapp PR, Morrison JH (2010). Selective changes in thin spine density and morphology in monkey prefrontal cortex correlate with aging-related cognitive impairment. The Journal of Neuroscience.

[bib14] Elston GN, Fujita I (2014). Pyramidal cell development: postnatal spinogenesis, dendritic growth, axon growth, and electrophysiology. Frontiers in Neuroanatomy.

[bib15] Erraji-Benchekroun L, Underwood MD, Arango V, Galfalvy H, Pavlidis P, Smyrniotopoulos P, Mann JJ, Sibille E (2005). Molecular aging in human prefrontal cortex is selective and continuous throughout adult life. Biological Psychiatry.

[bib16] Etherington SJ, Williams SR (2011). Postnatal development of intrinsic and synaptic properties transforms signaling in the layer 5 excitatory neural network of the visual cortex. The Journal of Neuroscience.

[bib17] Eyal G, Mansvelder HD, de Kock CPJ, Segev I (2014). Dendrites impact the encoding capabilities of the axon. The Journal of Neuroscience.

[bib18] Globus A, Rosenzweig MR, Bennett EL, Diamond MC (1973). Effects of differential experience on dendritic spine counts in rat cerebral cortex. Journal of Comparative and Physiological Psychology.

[bib19] Goldstein SAN, Bockenhauer D, O’Kelly I, Zilberberg N (2001). Potassium leak channels and the KCNK family of two-p-domain subunits. Nature Reviews Neuroscience.

[bib20] Greenough WT, Volkmar FR (1973). Pattern of dendritic branching in occipital cortex of rats reared in complex environments. Experimental Neurology.

[bib21] Guet-McCreight A, Chameh HM, Mahallati S, Wishart M, Tripathy SJ, Valiante TA, Hay E (2023). Age-dependent increased sag amplitude in human pyramidal neurons dampens baseline cortical activity. Cerebral Cortex.

[bib22] Holloway RL (1966). Dendritic branching: some preliminary results of training and complexity in rat visual cortex. Brain Research.

[bib23] Horibe S, Tarusawa E, Komatsu Y, Yoshimura Y (2014). Ni(2+)-sensitive T-type Ca(2+) channel currents are regulated in parallel with synaptic and visual response plasticity in visual cortex. Neuroscience Research.

[bib24] Huguenard JR, Hamill OP, Prince DA (1988). Developmental changes in Na+ conductances in rat neocortical neurons: appearance of a slowly inactivating component. Journal of Neurophysiology.

[bib25] Huttenlocher PR (1979). Synaptic density in human frontal cortex - developmental changes and effects of aging. Brain Research.

[bib26] Huttenlocher PR, de Courten C, Garey LJ, Van der Loos H (1982). Synaptogenesis in human visual cortex--evidence for synapse elimination during normal development. Neuroscience Letters.

[bib27] Huttenlocher PR, Dabholkar AS (1997). Regional differences in synaptogenesis in human cerebral cortex. The Journal of Comparative Neurology.

[bib28] Jacobs B, Driscoll L, Schall M (1997). Life-span dendritic and spine changes in areas 10 and 18 of human cortex: A quantitative Golgi study. The Journal of Comparative Neurology.

[bib29] Kalmbach BE, Buchin A, Long B, Close J, Nandi A, Miller JA, Bakken TE, Hodge RD, Chong P, de Frates R, Dai K, Maltzer Z, Nicovich PR, Keene CD, Silbergeld DL, Gwinn RP, Cobbs C, Ko AL, Ojemann JG, Koch C, Anastassiou CA, Lein ES, Ting JT (2018). h-channels contribute to divergent intrinsic membrane properties of supragranular pyramidal neurons in human versus mouse cerebral cortex. Neuron.

[bib30] Kang HJ, Kawasawa YI, Cheng F, Zhu Y, Xu X, Li M, Sousa AMM, Pletikos M, Meyer KA, Sedmak G, Guennel T, Shin Y, Johnson MB, Krsnik Z, Mayer S, Fertuzinhos S, Umlauf S, Lisgo SN, Vortmeyer A, Weinberger DR, Mane S, Hyde TM, Huttner A, Reimers M, Kleinman JE, Sestan N (2011). Spatio-temporal transcriptome of the human brain. Nature.

[bib31] Kasper EM, Larkman AU, Lübke J, Blakemore C (1994). Pyramidal neurons in layer 5 of the rat visual cortex. II. Development of Electrophysiological Properties. J. Comp. Neurol.

[bib32] Kastellakis G, Tasciotti S, Pandi I, Poirazi P (2023). The dendritic engram. Frontiers in Behavioral Neuroscience.

[bib33] Kelly AMC, Di Martino A, Uddin LQ, Shehzad Z, Gee DG, Reiss PT, Margulies DS, Castellanos FX, Milham MP (2009). Development of anterior cingulate functional connectivity from late childhood to early adulthood. Cerebral Cortex.

[bib34] Koenderink MJTh, Uylings HBM (1995). Postnatal maturation of layer V pyramidal neurons in the human prefrontal cortex: a quantitative Golgi analysis. Brain Research.

[bib35] Kroon T, van Hugte E, van Linge L, Mansvelder HD, Meredith RM (2019). Early postnatal development of pyramidal neurons across layers of the mouse medial prefrontal cortex. Scientific Reports.

[bib36] Kuzawa CW, Chugani HT, Grossman LI, Lipovich L, Muzik O, Hof PR, Wildman DE, Sherwood CC, Leonard WR, Lange N (2014). Metabolic costs and evolutionary implications of human brain development. PNAS.

[bib37] Li BZ, Sumera A, Booker SA, McCullagh EA (2023). Current best practices for analysis of dendritic spine morphology and number in neurodevelopmental disorder research. ACS Chemical Neuroscience.

[bib38] Linaro D, Vermaercke B, Iwata R, Ramaswamy A, Libé-Philippot B, Boubakar L, Davis BA, Wierda K, Davie K, Poovathingal S, Penttila PA, Bilheu A, De Bruyne L, Gall D, Conzelmann KK, Bonin V, Vanderhaeghen P (2019). Xenotransplanted human cortical neurons reveal species-specific development and functional integration into mouse visual circuits. Neuron.

[bib39] Luebke JI, Chang YM (2007). Effects of aging on the electrophysiological properties of layer 5 pyramidal cells in the monkey prefrontal cortex. Neuroscience.

[bib40] Luebke JI, Medalla M, Amatrudo JM, Weaver CM, Crimins JL, Hunt B, Hof PR, Peters A (2015). Age-related changes to layer 3 pyramidal cells in the rhesus monkey visual cortex. Cerebral Cortex.

[bib41] Luhmann HJ, Reiprich RA, Hanganu I, Kilb W (2000). Cellular physiology of the neonatal rat cerebral cortex: Intrinsic membrane properties, sodium and calcium currents. Journal of Neuroscience Research.

[bib42] Luna B, Marek S, Larsen B, Tervo-Clemmens B, Chahal R (2015). An integrative model of the maturation of cognitive control. Annual Review of Neuroscience.

[bib43] Mainen ZF, Sejnowski TJ (1996). Influence of dendritic structure on firing pattern in model neocortical neurons. Nature.

[bib44] Matsuzaki M, Ellis-Davies GC, Nemoto T, Miyashita Y, Iino M, Kasai H (2001). Dendritic spine geometry is critical for AMPA receptor expression in hippocampal CA1 pyramidal neurons. Nature Neuroscience.

[bib45] Mavroudis IA, Manani MG, Petrides F, Dados D, Ciobica A, Padurariu M, Petsoglou K, Njau SN, Costa VG, Baloyannis SJ (2015). Age-related dendritic and spinal alterations of pyramidal cells of the human visual cortex. Folia Neuropathologica.

[bib46] McAllister AK (2000). Cellular and molecular mechanisms of dendrite growth. Cerebral Cortex.

[bib47] McCormick DA, Prince DA (1987). Post-natal development of electrophysiological properties of rat cerebral cortical pyramidal neurones. The Journal of Physiology.

[bib48] Molineux ML, Fernandez FR, Mehaffey WH, Turner RW (2005). A-type and T-type currents interact to produce A novel spike latency-voltage relationship in cerebellar stellate cells. The Journal of Neuroscience.

[bib49] Molnár Z, Luhmann HJ, Kanold PO (2020). Transient cortical circuits match spontaneous and sensory-driven activity during development. Science.

[bib50] Moore TL, Medalla M, Ibañez S, Wimmer K, Mojica CA, Killiany RJ, Moss MB, Luebke JI, Rosene DL (2023). Neuronal properties of pyramidal cells in lateral prefrontal cortex of the aging rhesus monkey brain are associated with performance deficits on spatial working memory but not executive function. GeroScience.

[bib51] Moradi Chameh H, Rich S, Wang L, Chen FD, Zhang L, Carlen PL, Tripathy SJ, Valiante TA (2021). Diversity amongst human cortical pyramidal neurons revealed via their sag currents and frequency preferences. Nature Communications.

[bib52] Mrzljak L, Uylings HB, Van Eden CG, Judás M (1990). Neuronal development in human prefrontal cortex in prenatal and postnatal stages. Progress in Brain Research.

[bib53] Nusser Z, Lujan R, Laube G, Roberts JD, Molnar E, Somogyi P (1998). Cell type and pathway dependence of synaptic AMPA receptor number and variability in the hippocampus. Neuron.

[bib54] Oláh G, Lákovics R, Shapira S, Leibner Y, Szűcs A, Csajbók ÉA, Barzó P, Molnár G, Segev I, Tamás G (2024). Accelerated signal propagation speed in human neocortical microcircuits. eLife.

[bib55] Oswald AMM, Reyes AD (2011). Development of inhibitory timescales in auditory cortex. Cerebral Cortex.

[bib56] Perez-García P, Pardillo-Díaz R, Geribaldi-Doldán N, Gómez-Oliva R, Domínguez-García S, Castro C, Nunez-Abades P, Carrascal L (2021). Refinement of active and passive membrane properties of layer V pyramidal neurons in rat primary motor cortex during postnatal development. Frontiers in Molecular Neuroscience.

[bib57] Petanjek Z, Judas M, Kostović I, Uylings HBM (2008). Lifespan alterations of basal dendritic trees of pyramidal neurons in the human prefrontal cortex: A layer-specific pattern. Cerebral Cortex.

[bib58] Petanjek Z, Judaš M, Šimic G, Rasin MR, Uylings HBM, Rakic P, Kostovic I (2011). Extraordinary neoteny of synaptic spines in the human prefrontal cortex. PNAS.

[bib59] Petanjek Z, Sedmak D, Džaja D, Hladnik A, Rašin MR, Jovanov-Milosevic N (2019). The protracted maturation of associative layer IIIC pyramidal neurons in the human prefrontal cortex during childhood: a major role in cognitive development and selective alteration in autism. Frontiers in Psychiatry.

[bib60] Peters R (2006). Ageing and the brain. Postgraduate Medical Journal.

[bib61] Picken Bahrey HL, Moody WJ (2003). Early development of voltage-gated ion currents and firing properties in neurons of the mouse cerebral cortex. Journal of Neurophysiology.

[bib62] Popescu IR, Le KQ, Ducote AL, Li JE, Leland AE, Mostany R (2021). Increased intrinsic excitability and decreased synaptic inhibition in aged somatosensory cortex pyramidal neurons. Neurobiology of Aging.

[bib63] Rakic P (2009). Evolution of the neocortex: a perspective from developmental biology. Nature Reviews. Neuroscience.

[bib64] Stiles J, Jernigan TL (2010). The basics of brain development. Neuropsychology Review.

[bib65] van Blooijs D, van den Boom MA, van der Aar JF, Huiskamp GM, Castegnaro G, Demuru M, Zweiphenning W, van Eijsden P, Miller KJ, Leijten FSS, Hermes D (2023). Developmental trajectory of transmission speed in the human brain. Nature Neuroscience.

[bib66] van Elburg RAJ, van Ooyen A (2010). Impact of dendritic size and dendritic topology on burst firing in pyramidal cells. PLOS Computational Biology.

[bib67] Vardalaki D, Chung K, Harnett MT (2022). Filopodia are a structural substrate for silent synapses in adult neocortex. Nature.

[bib68] Vetter P, Roth A, Häusser M (2001). Propagation of action potentials in dendrites depends on dendritic morphology. Journal of Neurophysiology.

[bib69] Wang M, Ramos BP, Paspalas CD, Shu Y, Simen A, Duque A, Vijayraghavan S, Brennan A, Dudley A, Nou E, Mazer JA, McCormick DA, Arnsten AFT (2007). Alpha2A-adrenoceptors strengthen working memory networks by inhibiting cAMP-HCN channel signaling in prefrontal cortex. Cell.

[bib70] Wang M, Gamo NJ, Yang Y, Jin LE, Wang X-J, Laubach M, Mazer JA, Lee D, Arnsten AFT (2011). Neuronal basis of age-related working memory decline. Nature.

[bib71] Yeatman JD, Wandell BA, Mezer AA (2014). Lifespan maturation and degeneration of human brain white matter. Nature Communications.

[bib72] Yoshimura Y, Dantzker JLM, Callaway EM (2005). Excitatory cortical neurons form fine-scale functional networks. Nature.

[bib73] Zhang Z (2004). Maturation of layer V pyramidal neurons in the rat prefrontal cortex: intrinsic properties and synaptic function. Journal of Neurophysiology.

[bib74] Zhu JJ (2000). Maturation of layer 5 neocortical pyramidal neurons: amplifying salient layer 1 and layer 4 inputs by Ca2+ action potentials in adult rat tuft dendrites. The Journal of Physiology.

